# High level secretion of cellobiohydrolases by *Saccharomyces cerevisiae*

**DOI:** 10.1186/1754-6834-4-30

**Published:** 2011-09-12

**Authors:** Marja Ilmén, Riaan den Haan, Elena Brevnova, John McBride, Erin Wiswall, Allan Froehlich, Anu Koivula, Sanni P Voutilainen, Matti Siika-aho, Daniël C la Grange, Naomi Thorngren, Simon Ahlgren, Mark Mellon, Kristen Deleault, Vineet Rajgarhia, Willem H van Zyl, Merja Penttilä

**Affiliations:** 1 VTT Technical Research Centre of Finland, Tietotie 2, Espoo, FI-02044 VTT, Finland; 2 Department of Microbiology, University of Stellenbosch, De Beer Street, Stellenbosch 7600, South Africa; 3 Mascoma Corporation, 67 Etna Road, Suite 300, Lebanon, NH 03766, USA; 4 Total Gas & Power, 5858 Horton Street, Suite 253, Emeryville, CA 94608, USA

**Keywords:** biofuels, cellulolytic yeast, UPR

## Abstract

**Background:**

The main technological impediment to widespread utilization of lignocellulose for the production of fuels and chemicals is the lack of low-cost technologies to overcome its recalcitrance. Organisms that hydrolyze lignocellulose and produce a valuable product such as ethanol at a high rate and titer could significantly reduce the costs of biomass conversion technologies, and will allow separate conversion steps to be combined in a consolidated bioprocess (CBP). Development of *Saccharomyces cerevisiae *for CBP requires the high level secretion of cellulases, particularly cellobiohydrolases.

**Results:**

We expressed various cellobiohydrolases to identify enzymes that were efficiently secreted by *S. cerevisiae*. For enhanced cellulose hydrolysis, we engineered bimodular derivatives of a well secreted enzyme that naturally lacks the carbohydrate-binding module, and constructed strains expressing combinations of *cbh1 *and *cbh2 *genes. Though there was significant variability in the enzyme levels produced, up to approximately 0.3 g/L CBH1 and approximately 1 g/L CBH2 could be produced in high cell density fermentations. Furthermore, we could show activation of the unfolded protein response as a result of cellobiohydrolase production. Finally, we report fermentation of microcrystalline cellulose (Avicel™) to ethanol by CBH-producing *S. cerevisiae *strains with the addition of beta-glucosidase.

**Conclusions:**

Gene or protein specific features and compatibility with the host are important for efficient cellobiohydrolase secretion in yeast. The present work demonstrated that production of both CBH1 and CBH2 could be improved to levels where the barrier to CBH sufficiency in the hydrolysis of cellulose was overcome.

## Background

The baker's yeast *Saccharomyces cerevisiae *has been extensively studied as a production host for heterologous proteins and other valuable compounds [1-3]. Due to its long use and beneficial properties as a robust production host in large scale, especially commercial ethanol production, interest in metabolic engineering and utilization of the engineered *S. cerevisiae *in the production of fuels and other bulk chemicals from renewable resources keeps increasing. *S. cerevisiae *is expected to continue as a prominent host in future biorefineries that aim to effectively convert currently unutilized plant materials to useful products.

A low-cost bioprocess to produce bulk fuels and chemicals requires several changes to be made in the metabolism of *S. cerevisiae*. One of these is the utilization and fermentation of all biomass derived sugars. Consequently, engineering *S. cerevisiae *for pentose sugar fermentation, particularly D-xylose and L-arabinose derived from lignocellulosic raw materials, has been one of the successfully met challenges in the development of second generation bioethanol production (reviewed in [4]). Lignocellulose hydrolysis to fermentable sugars is currently achieved by biomass pretreatment and the addition of separately produced enzyme preparations into the process. The enzymes are often also allowed to act during the actual fermentation leading to simultaneous saccharification and fermentation (SSF) [5,6]. While SSF has benefits such as uptake of the released glucose by the fermenting organism, which counteracts glucose inhibition of cellulases, the high cost of added enzymes is still a major factor in the process economics. Since the conversion of lignocellulosic raw material into monomer sugars is limited by the rate and extent of conversion of the plant polysaccharides by enzymes, engineering of yeast to secrete rate limiting enzymes would complement extensive efforts undertaken to engineer existing enzymes, and further streamline the process towards a consolidated bioprocessing (CBP) and lower production costs [6,7].

Hydrolysis of cellulose, in particular the complete hydrolysis of the more crystalline parts, is considered a key challenge in biomass hydrolysis [8,9]. Cellulose is hydrolyzed with mixtures of three different types of enzymes that hydrolyze the β-1,4-glycosidic bonds, cellobiohydrolases (CBHs, EC 3.2.1.91), endo-β-1,4-glucanases (EG, EC 3.2.1.4) and β-glucosidase (EC 3.2.1.21) (for review see [5]). CBHs are instrumental in the hydrolysis of natural cellulose that contains highly ordered crystalline regions. These enzymes act from cellulose chain ends, releasing mainly cellobiose. The endoglucanases attack more amorphous parts and hydrolyze cellulose from the middle of the chains, acting in synergy with CBHs to hydrolyze the substrate to small oligosaccharides. Finally, β-glucosidase hydrolyses cello-oligosaccharides to glucose.

The most studied cellulolytic fungus, *Trichoderma reesei*, produces up to about 80% of the total secreted protein as CBH, and the best production strains can secrete tens of grams per liter of these enzymes [10]. There are two major fungal CBH classes, separated into the glycosyl hydrolase families GH-7 (also called CBH1) and GH6 (CBH2) based on their sequence similarity and predicted structural and functional relationships (http://www.cazy.org/; [11]). The catalytic domains of these two enzyme classes are structurally different but both share a tunnel-like active site. Many fungal CBHs have a separate, small cellulose-binding module (CBM) belonging to the CBM-1 family http://www.cazy.org/. In GH-7 CBHs, the CBM is attached to the C-terminus via a flexible linker, and in GH-6 enzymes to the N-terminus. The CBM is considered to be essential for hydrolysis of crystalline cellulose [12]. Several studies indicate that CBH1 and CBH2 types of enzymes also act in synergy in cellulose hydrolysis [8,12,13].

Cellulases were among the first heterologous proteins expressed in yeast [14-17] and since then several reports have shown that *S. cerevisiae *can secrete fungal hydrolytic enzymes, including CBHs. The naturally *N*- and *O*-glycosylated CBH enzymes are typically hyperglycosylated with high mannose glycans in *S. cerevisiae *[15,18-20]. The examples of CBH expressed in yeast include CBH1 (Cel7A) and CBH2 (Cel6A) of *T. reesei *[15,19,21,22], and CBHs of other fungi [18-20,23,24]. Although the enzymes retain activity when expressed in *S. cerevisiae*, there are results indicating that the activity of the yeast-produced enzymes is impaired in comparison to the native proteins, which in some cases could be due to overglycosylation [15,23,25] or misfolding [26].

Relatively high protein production levels of 1-10% of cellular protein have been reported in *S. cerevisiae *[27]. However, there have also been reports of poor levels of protein secretion, especially when expressing cellulase-encoding genes [19]. In order to maximize production of a heterologous protein, the gene copy number, codon usage and the choice of promoters are important for obtaining sufficient transcript levels in the host. Subsequently, stable transcripts are required to maintain high levels of translation of the heterologous gene. Production of secreted proteins also requires that they are able to enter the secretory pathway, are correctly folded and processed there, and finally are secreted in an active form into the extracellular medium. Heterologous protein production is known to be limited by cellular stress reactions that can largely influence productivity [28]. For example, the accumulation of unfolded proteins in the endoplasmic reticulum (ER) causes stress and induces the unfolded protein response (UPR) that coordinates the physiological responses to ER stress [29]. It is well established that Hac1p mediates the UPR in *S. cerevisiae *[30,31]. The constitutively synthesized *HAC1 *mRNA is spliced in response to ER stress, resulting in the synthesis of the active DNA-binding transcription factor Hac1p. This activates the expression of genes coding for chaperones, foldases and components of the ER associated degradation system in order to adapt to the situation by increasing the protein folding capacity of the ER and by clearing misfolded proteins from the ER [32]. Moreover, autoregulation of the *HAC1 *gene is required for sustained activation of the UPR and resistance to ER stress [33].

In this work we carried out a comprehensive study of expression of various *cbh *genes to identify enzymes that can be efficiently secreted by *S. cerevisiae *in an active form into extracellular medium, which is fundamental for a successful CBP or SSF process. With the aim of enhancing cellulose hydrolysis further from the levels obtained during screening, we engineered bimodular derivatives of a well secreted CBM-less enzyme and constructed strains expressing various combinations of the *cbh1 *and *cbh2 *genes. We examined the physiological impact of CBH production in *S. cerevisiae *expressing the different *cbh *genes and discovered correlations with the UPR. Finally, we report fermentation of microcrystalline Avicel^™ ^cellulose to ethanol by selected CBH-expressing *S. cerevisiae *strains with the aid of externally added β-glucosidase.

## Results

### Expression and secretion of CBH1 and CBH2

To identify enzymes that are efficiently secreted in an active form into the culture supernatant, we screened 14 *cbh1 *(Cel7A) and 10 *cbh2 *(Cel6A) genes from ascomycetes by functional expression in *S. cerevisiae*. Candidate fungal *cbh1 *genes (Table 1) were synthesized with *S. cerevisiae *codon bias and expressed under the control of the enolase gene (*ENO1*) promoter and terminator on an *URA3 *selectable episomal multicopy vector to ensure the high expression level needed for addressing secretability. The *cbh2 *genes were expressed under the control of the 3-phosphoglycerate kinase gene (*PGK1*) promoter and terminator. The genes contained either their native signal sequence for secretion, the *S. cerevisiae *mating factor α-1 precursor secretion signal or the *T. reesei xyn2 *signal sequence (Table 1). To create autoselective strains, the *FUR1 *gene encoding a uracil phosphoribosyltransferase that converts uracil to uridine monophosphate was disrupted to ensure plasmid maintenance in non-selective conditions. All the *cbh*-expressing and the empty vector control strains were grown in yeast extract peptone dextrose (YPD) medium, and a subset of selected strains were also grown in defined soybean casein digest without uracil (SCD^-URA^) medium (pH 6) with 2% glucose as the carbon source. Samples were taken for the determination of CBH activity.

**Table 1:** Cellobiohydrolases expressed in *S. cerevisiae *in this study

Species and gene name	GenBank accession number	Expression plasmid	Recombinant yeast strain abbreviation
*cbh1 *encoding genes: expressed under transcriptional control of *S. cerevisiae ENO1 *promoter			
*Humicola grisea cbh1*^a, b^	[GenBank:CAA35159]	pRDH103	*Sc*[*H.g.cbh1*]
*Thermoascus aurantiacus cbh1*^a, b^	[GenBank:AAL83303]	pRDH104	*Sc*[*T.a.cbh1*]
*Talaromyces emersonii cbh1*^a, b^	[GenBank:AAL89553]	pRDH105	*Sc*[*T.e.cbh1*]
*Neosartorya fischeri cbh1*^c, d^	[GenBank:XP_001258278]	pRnD317	*Sc*[*N.f.cbh1*]
*Penicillium janthinellum cbh1*^c, d^	[GenBank:X59054.1]	pRnD353	*Sc*[*P.j.cbh1*]
*Gibberella zeae cbh1*^c, d^	[GenBank:AY196784.2]	pRnD318	*Sc*[*G.z.cbh1*]
*Nectria haematococca cbh1*^c, d^	[GenBank:AY502070.1]	pRnD319	*Sc*[*N.h.cbh1*]
*Fusarium poae cbh1*^c, d^	[GenBank:AY706934]	pRnD320	*Sc*[*F.p.cbh1*]
*Aspergillus terreus cbh1*^c, d^	[GenBank:XM_001214180]	pRnD322	*Sc*[*As.t.cbh1*]
*Penicillium chrysogenum cbh1*^c, d^	[GenBank:AY790330]	pRnD323	*Sc*[*P.c.cbh1*]
*Neurospora crassa cbh1*^c, d^	[GenBank:X77778]	pRnD324	*Sc*[*N.c.cbh1*]
*Chaetomium thermophilum cbh1^b, e^*	[GenBank:CAM98448.1]	pMI569	*Sc*[*C.t.cbh1*]
*Acremonium thermophilum cbh1^b, e^*	[GenBank:CAM98445.1]	pMI567	*Sc*[*Ac.t.cbh1*]
*Trichoderma reesei cbh1^f^*	[SwissProt::P62694.1]	pRDH101	*Sc*[*T.r.cbh1*]

*cbh2 *encoding genes: expressed under transcriptional control of *S. cerevisiae PGK1 *promoter			
*Cochliobolus heterostrophus *C4 *cel7^b, c^*	[GenBank:AAM76664.1]	pRDH150	*Sc*[*C.h.cbh2*]
*Gibberella zeae K59 cel6 ^b, c^*	[GenBank:AAQ72468.1]	pRDH151	*Sc*[*G.z.cbh2*]
*Irpex lacteus MC-2 cex ^b, c^*	[GenBank:BAG48183.1]	pRDH152	*Sc*[*I.l.cbh2*]
*Volvariella volvacea cbhII-I ^b, c^*	[GenBank:AAT64008.1]	pRDH153	*Sc*[*V.v.cbh2*]
*Piromyces sp. E2 cel6A ^b, c^*	[GenBank:AAL92497.1]	pRDH154	*Sc*[*P*.sp.*cbh2*]
*Talaromyces emersonii cbh2^a, b^*	[GenBank:AF439936]	pRDH106	*Sc*[*T.e.cbh2*]
*Trichoderma reesei cbh2^a, f^*	[SwissProt:P07987.1]	pRDH107	*Sc*[*T.r.cbh2*]
*Chrysosporium lucknowense cbh2b^b, d^*	[EMBL-Bank::HH793136.1]	pMI574	*Sc*[*C.l.cbh2b*]; M0969^g^
*Acremonium cellulolyticus cbh2^b, e^*	[SwissProt:O93837]	pMI571	*Sc*[*A.c.cbh2*]
*Chaetomium thermophilum cbh2^b, e^*	[SwissProt:Q5G2D4]	pMI573	*Sc*[*C.t.cbh2*]

*^a^*Synthesized by GenScript (Piscataway, NJ, USA); ^b^native secretion signal; ^c^synthesized by Geneart (Regensburg, Germany); ^d^*S. cerevisiae *mating factor α-1 precursor secretion signal; ^e^synthesized by Codon Devices (Cambridge, UK); ^f^*Trichoderma reesei xyn2 *secretion signal; ^g^diploid *ura3Δ/ura3Δ FUR1/fur1Δ *strain which has a functional xylose pathway, *i.e*. over expressed pentose pathway genes and *Piromyces xylA*, and *gre3 *deleted.

The first screening for CBH production in YPD cultivations was carried out using two enzymatic methods. To assess enzymatic hydrolysis of polymeric insoluble cellulose, secreted CBH1 and CBH2 activity was measured by incubating the cell-free yeast culture supernatants with Avicel PH105 cellulose in the presence of β-glucosidase (Novozyme 188) to hydrolyze the cellobiose released from cellulose to glucose, followed by determination of the reducing sugars formed. CBH1 production was also measured by activity on the soluble fluorescent substrate 4-methylumbelliferyl β-D-lactoside (MULac); an analogous substrate is not available for CBH2. The activity of the highly expressed *T.e*.CBH1 reached its maximum after three days growth on YPD medium and therefore day three samples were analyzed for all strains.

**Figure 1:**
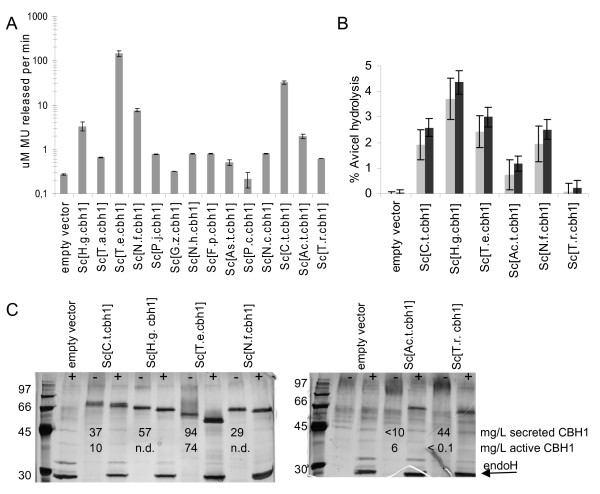
**Production of CBH1 enzymes**. **(a) **Secreted MULac activity (microM MU released per minute) produced by recombinant strains expressing *cbh1 *genes cultured in YPD medium for three days. **(b) **Percentage Avicel hydrolysis by supernatants of the same strains in 24 (grey bars) and 48 (black bars) hours. The values shown are the mean values of three repeats ± standard deviation. **(c) **Reducing 12% SDS-PAGE of cell free yeast culture supernatants (20 μL) visualized by silver staining. Samples were either deglycosylated with endoH (+) or non-treated (-). Molecular weight markers (97, 66, 45, 30 kDa) are shown on the left. The concentration of total secreted protein (mg/L) as determined by the BioRad protein assay, and the concentration of active CBH1 (mg/L) estimated based on the MULac activity, are indicated by numbers.

The CBH1 activities measured in the cell-free culture supernatants on MULac ranged over two to three orders of magnitude (Figure 1A): the catalytic activity against MULac in *T.e*.CBH1-containing supernatants was at least 100-fold higher than that in nine other strains' supernatants tested. Because the hydrolytic efficiencies on MULac and on insoluble cellulose often differ between the different enzymes, all enzymes were assayed on both substrates. The top five strains expressed *cbh1 *genes of *Talaromyces emersonii, Humicola grisea, Neosartorya fischeri, Chaetomium thermophilum*, or *Acremonium thermophilum *which resulted in clearly detectable responses in both activity assays (Figure 1 and Additional file 1).

Results of CBH1 production by a subset of the best strains and reference strains grown in SCD^-URA ^medium were consistent with the results in YPD medium with regard to activity-based ranking of the best strains. The culture supernatant of *Sc*[*T.e.cbh1*] had by far the highest activity on the soluble substrate MULac in both media (Figure 1A and Additional file 1), while *Sc*[*H.g.cbh1*] had relatively low activity on MULac, even though it had the highest activity on crystalline cellulose (Figure 1B and Additional file 1). Measurement of protein concentration in the cell-free SCD^-URA ^culture supernatants and SDS-PAGE analyses confirmed that *T.e*.CBH1 enzyme was abundantly produced relative to the other CBH1 enzymes (Figure 1C). All CBH1 proteins contained *N*-glycans, as their mobility in the gel was altered following enzymatic *N*-glycan removal by endoH treatment (Figure 1C) which shows that hyperglycosylation of the CBH1 enzymes occurs in yeast. Avicel conversion by *Ac.t*.CBH1 and *T.r*.CBH1 was the least efficient and the enzymes could be visualized as distinct bands only after the removal of *N*-linked glycans followed by SDS-PAGE (Figure 1C). Even after the removal of *N*-glycans all the protein bands had a larger molecular weight than predicted based on the amino acid sequence, as could be expected based on several earlier studies. For example, it is likely that *O*-glycosylation of the CBH1 enzymes takes place in *S. cerevisiae *as it does in the native organisms.

Since the CBH1 enzyme appeared in most strains as the major band in the SCD^-URA ^culture supernatant in SDS-PAGE, and the level of host proteins secreted in these conditions was not noticeably changed as a result of *cbh1 *expression, it seemed reasonable to estimate the amount of secreted CBH1 by measuring the protein concentrations with the BioRad protein assay in the cell-free culture supernatants and subtracting the values for the empty vector control from those of the CBH-producing strains. Estimations of *T.e*.CBH1 protein concentration based on total protein and the concentration of active *T.e*.CBH1 based on specific activity on MULac (Figure 1C) were fairly consistent, as they were for the *Ac.t*.CBH1, which was produced at a low level but was evidently capable of cellulose conversion. In comparison, the enzymatic activity of the *T. reesei *CBH1 was not proportional to the amount of protein measurement (Figure 1C), suggesting that only a small fraction of the secreted enzyme pool was enzymatically active, similarly to the *T.r*.CBH1 expressed in *Pichia pastoris *[26]. The concentration of active *C.t*.CBH1 also was lower than the concentration estimated from the secreted protein measurement (Figure 1C).

**Figure 2:**
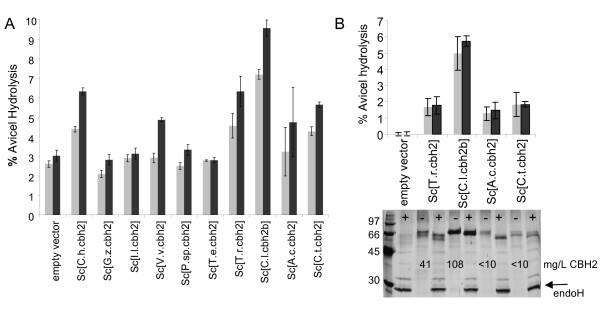
**Production of CBH2 enzymes**. Percentage of Avicel hydrolysis by yeast culture supernatants grown in **(a) **YPD medium or **(b) **in SCD^-URA ^medium (pH 6) for 3 days. The assay incubation time was 24 (grey bars) and 48 (black bars) hours. The values shown are the mean values of three repeats ± standard deviation. Reducing 12% SDS-PAGE of cell free yeast culture supernatants (20 μL) visualized by silver staining. Samples were either deglycosylated with endoH (+) or non-treated (-). The concentration of total secreted protein (mg/L) as determined by the BioRad protein assay is indicated.

Ten *cbh2 *genes (Table 1) were synthesized with *S. cerevisiae *codon bias and expressed under the control of the *PGK1 *promoter and terminator on a *URA3 *selectable episomal multicopy vector. Their activity in Avicel hydrolysis was studied as above. The culture supernatants of *Sc*[*C.l.cbh2b*] showed superior Avicel conversion ability both in YPD and in SCD^-URA ^medium (Figure 2). Protein concentration in the cell-free SCD^-URA ^culture supernatants and SDS-PAGE analysis also showed clearly that *C.l*.CBH2b was by far the most abundantly produced CBH2 enzyme (Figure 2B).

### Improvement of cellulose hydrolysis by CBM attachment to CBH1

The fact that *T.e*.CBH1 is secreted well but lacks a CBM raised the possibility of improving the enzyme by adding a CBM to the catalytic domain. Three different constructs were designed, in which the linker and CBM originating from *H.g.cbh1, T.r.cbh1 *or *C.t.cbh1 *were fused to the C-terminus of the *T.e.cbh1 *(Table 2 and Additional file 2). The CBMs have high overall identity but there are differences in the aromatic amino acids predicted to contact cellulose (Additional file 2) and in the disulfide bridge formation; the *H. grisea *and *T. reesei cbh1 *CBMs have four cysteines whereas the *C. thermophilum cbh1 *CBM has six cysteines. Furthermore, the linkers differ in length and amino acid sequence as well as in the glycosylation pattern; all the linkers are rich in serine and threonine but the number of possible *O*-glycosylation target sites differs. The *Te*CBH1-*Tr*CBM-C enzyme has an additional *N*-glycosylation target site lacking from the other two bimodular enzymes. In a fourth construct the linker and CBM of *T.r.cbh2 *were fused to the N-terminus of *T.e.cbh1*. In addition, two variants with different signal sequences for secretion were constructed for the N-terminal fusion and for the C-terminal fusion with *T.r.cbh1 *CBM, one with the *T. emersonii cbh1 *signal sequence and the other with the *T. reesei xyn2 *signal sequence for secretion (Table 2).

**Table 2:** Fusion genes created with *T. emersonii cbh1 *for expression in *S. cerevisiae*

Origin of CBM	Position attached	Expression plasmid	Recombinant yeast strain abbreviation	Primers used for construction (5'-3')
*T. reesei cbh1^a^*	C-terminus	pMI529	*Sc*[*Tecbh1-TrCBM-C*]; M0759^c^	399Trcbh1-L GCGACGAGTCAACCCTCCAGGTGGTAACAGAGGTACTACCAC 400Trcbh1-R GCGACTCGAGGGCGCGCCTACAAACATTGAGAGTAGTATGGGTTTA
*H. grisea cbh1^a^*	C-terminus	pTeHg	*Sc*[*Tecbh1-HgCBM-C*]	Te-CBH-F TATAGAATTCTTAATTAAATGCTAAGAAGAGCTTTACTATTG Te-CBH-R TATACGTCTCTGGACCGAATTTAATGTTGGAGTA Hg-CBM-F TATACGTCTCGGTCCAATCGGTTCCACAGT Hg-CBM-R TATACTCGAGGCGCGCCTTATAAACATTGAGAGTACCAGTC
*C. thermophilum cbh1^a^*	C-terminus	pMI566	*Sc*[*Tecbh1-CtCBM-C*]	392ENO1p-F CAGGATCCCAATTAATGTGAGTTACC 393TeCt-R ACAGTGGATCCGATTGGACCGAATTTAATGTTGG
*T. reesei cbh2^a^*	N-terminus	pMI528	*Sc*[*Tecbh1-TrCBM-N*]	406TEM CBH1 NCBM-L CCTCCGAATTCATGCTAAGAAGAGCTTTACTATTGA-GCTCTTCTGCTATCTTGGCCGTTAAGGCTCAAGCCTGCTCCTCTGTTTGG 407TEM CBH1 NCBM-R AAACTTCAAGTCACGTGGACATTGAGAGTCACAG
*T. reesei cbh2^b^*	N-terminus	pDLG117	*Sc*[*Tecbh1-TrCBM-N2*]	NCBM-L GAATTCATAATGGTCTCCTTC NCBM-R AAAGCTCTCGAGTTAAGAAGC NCBM-OL2 CGGTACCGGCTTGTTGAGAGTAAGTAGCAGTACCGG
*T. reesei cbh1^b^*	C-terminus	pDLG118	*Sc*[*Tecbh1-TrCBM-C2*]	CCBM-L GAATTCATAATGGTCTCCTTC CCBM-R AATCAAAAGCTCTCGAGTTAC CCBM-OL2n GTTACCACCTGGAGGGTTAGAAGCAGTGAAAGTGGAG

*^a ^*Native secretion signal; ^b^*T. reesei xyn2 *secretion signal; ^c^diploid *ura3Δ/ura3Δ FUR1/fur1Δ *strain which has a functional xylose pathway *i.e*. over expressed pentose pathway genes and *Piromyces xylA*, and *gre3 *deleted.

**Figure 3:**
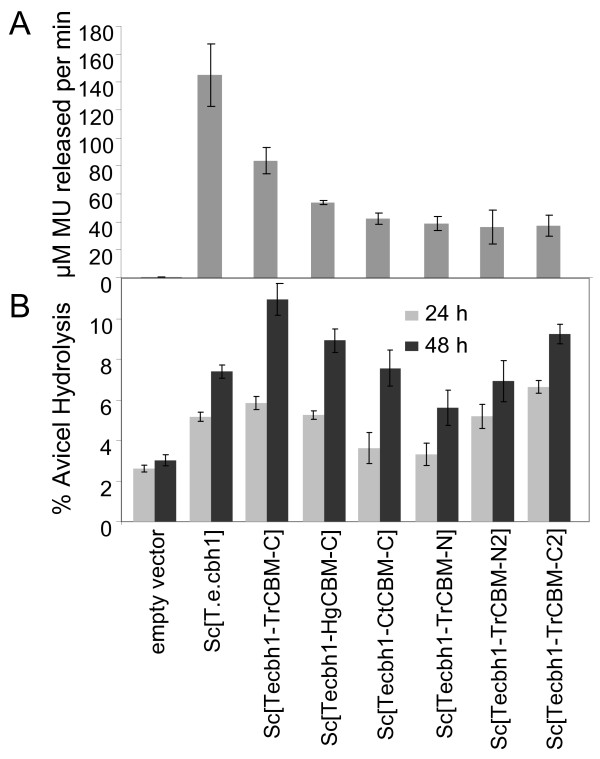
**Production of the bi-modular derivatives of *T.e*.CBH1 with the *T. reesei, C. thermophilum *or *H. grisea *linker-CBM sequences attached to the C-terminus**. Secreted activity on **(a) **MULac, and **(b) **Avicel hydrolyzed by supernatants of strains expressing the bimodular enzymes. The values shown are the mean values of three repeats ± standard deviation.

Data on CBH1 production as measured by MULac from the cell-free culture supernatant indicated that each of the fusion proteins was produced in an enzymatically active form even though the production level was reduced relative to *Sc*[*T.e.cbh1*] (Figure 3A). The C-terminal fusions appeared to perform better in the Avicel hydrolysis than the N-terminal fusions, as may have been expected based on the natural positioning of the CBMs in CBH1 enzymes. In spite of the reduction in the secreted enzyme concentration, Avicel conversion by equal volumes of the yeast culture supernatants containing the fusion between *T.e*.CBH1 and *T.r*.CBH1 CBM (Figure 3B) exceeded that of the non-fused protein, indicating that the CBM engineering was a useful strategy to enhance cellulose hydrolysis. It was repeatedly observed that the different fusion proteins were secreted at different levels, suggesting that the choice of the fusion partner or the design of the fusion can have a large effect on the levels of secreted protein. Yeast expression seemed also to affect the specific activity and proper folding of the purified fusion proteins (Voutilainen *et al*. unpublished results), further demonstrating the importance of choosing the right fusion partner. In a process where the extent of hydrolytic activity per volume in the yeast culture supernatant is important, as it is in a CBP process, the *Te*CBH1-*Tr*CBM-C appeared as the best fusion and was chosen for further studies.

### Co-secretion of CBH1 and CBH2

Because CBH1 and CBH2 act synergistically in the hydrolysis of crystalline cellulose [12], and they are believed to hydrolyze the cellulose chain from different ends, we chose potentially useful *cbh1 *and *cbh2 *genes and constructed strains expressing the two genes in ten different combinations (Table 3) to enhance cellulose hydrolysis. The *cbh1 *and *cbh2 *expression cassettes used above were cloned into the same 2-micron plasmid and CBH activities were analyzed from cell-free culture supernatants as above.

**Table 3:** Combinations of cellobiohydrolases expressed in *S. cerevisiae *in this study

*cbh1 *(under *ENO1*p/t)	*cbh2 *(under *PGK1*p/t)	Expression plasmid	Recombinant yeast strain abbreviation
*T. emersonii cbh1^a^*	*T. reesei cbh2 ^b^*	pRDH109^c^/pMI578^d^	Sc[*Tecbh1 & Trcbh2*]
*T. emersonii cbh1-T.r.CBM-C ^a^*	*T. reesei cbh2 ^b^*	pMI553^d^/pRDH125^c^	*Sc*[*Tecbh1-TrCBM-C & Trcbh2*]
*C. thermophilum cbh1 ^a^*	*T. reesei cbh2 ^b^*	pMI579^d^	*Sc*[*Ctcbh1 & Trcbh Clcbh2b*]
*H. grisea cbh1 ^a^*	*T. reesei cbh2 ^b^*	pRDH118^c^/pMI577^d^	*Sc*[*Hgcbh1 & Trcbh2*]
*T. aurantiacus cbh1 ^a^*	*T. reesei cbh2 ^b^*	pRDH120^c^	*Sc*[*Tacbh1 & Trcbh2*]
*T.r.CBM-N-T. emersonii cbh1^b^*	*T. reesei cbh2 ^b^*	pRDH123^c^	*Sc*[*Tecbh1-TrCBM-N & Trcbh2*]
*T. emersonii cbh1 ^a^*	*C. lucknowense cbh2b ^a^*	pMI581^d^	*Sc*[*Tecbh1 & Clcbh2b*]
*C. thermophilum cbh1 ^a^*	*C. lucknowense cbh2b ^a^*	pMI583^d^	*Sc*[*Ctcbh1 & Clcbh2b*]
*T. emersonii cbh1-T.r.CBM ^a^*	*C. lucknowense cbh2b ^a^*	pRDH138^c^/pMI580^d^	*Sc*[*Tecbh1-TrCBM-C &Clcbh2b*]
*H. grisea cbh1 ^a^*	*C. lucknowense cbh2b ^a^*	pRDH140^c^/pMI582^d^	*Sc*[*Hgcbh1 & Clcbh2b*]

*^a ^*Native signal sequence; ^b ^*Trichoderma reesei xyn2 *signal sequence; ^c ^The *ENO1*p*-cbh1-ENO1*t and *PGK1*p*-cbh2-PGK1*t expression cassettes are oriented head to tail; ^d^ The *ENO1*p*-cbh1-ENO1*t and *PGK1*p*-cbh2-PGK1*t expression cassettes are oriented tail to tail.

**Figure 4:**
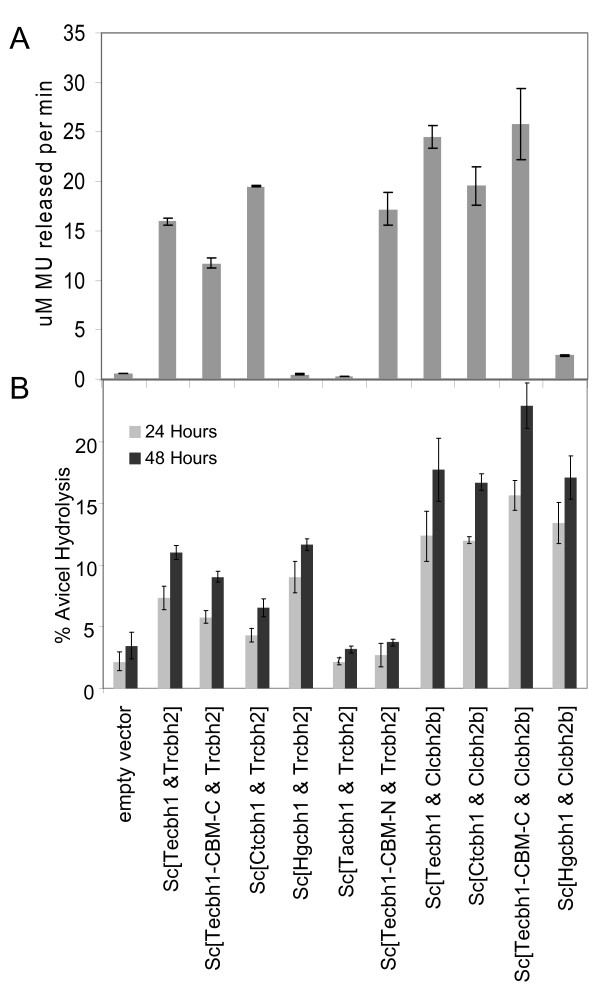
**Secreted CBH activity produced by recombinant strains co-expressing *cbh1 *and *cbh2 *genes cultured in YPD medium for three days**. **(a) **Secreted MULac activity (microM MU released per minute) and **(b) **Percentage of Avicel hydrolysis by supernatants of the same strains in 24 and 48 hours. The values shown are the mean values of three repeats ± standard deviation.

The efficiency of Avicel conversion to soluble sugars by the cell-free culture supernatants of *Sc*[*cbh1&cbh2*] strains exceeded that of the corresponding strains expressing only one enzyme in most cases. Co-expression of each of the four *cbh1 *genes, *T.e.cbh1, Tecbh1-TrCBM-C, H.g.cbh1 *and *C.t.cbh1*, with *C.l.cbh2b *resulted in a large increase in cellulose hydrolysis relative to the performance of the individual enzymes. The most successful combination, *Sc*[*TeCBH1-TrCBM-C & C.l.cbh2b*] resulted in 23% Avicel conversion (Figure 4), while the corresponding *Sc*[*TeCBH1-TrCBM-C*] and *Sc*[*C.l.cbh2b*] strains each achieved approximately 10% conversion in 48 hours, even though the CBH1 activity on MULac for *Sc*[*TeCBH1-TrCBM-C & C.l.cbh2b*] was lower than that for *Sc*[*TeCBH1-TrCBM-C*]. This suggests that the two enzymes acted in synergy in this environment. Activities on MULac also show that *T.e*.CBH1, *Te*CBH1-*Tr*CBM-C and *H.g*.CBH1 were more abundantly produced when co-expressed with *C.l.cbh2b *than with *T.r.cbh2 *(Figure 4A, Additional file 3A), which is an obvious cause for the observed differences in Avicel conversion (Figure 4B, Additional file 3B) in addition to the *cbh2 *itself. Furthermore, co-expression of *C.l.cbh2b *with any of the *cbh1 *genes yielded less CBH1 activity compared with strains expressing the corresponding *cbh1 *alone, although the extent of the effect varied between the enzyme combinations (Additional file 3A).

Two of the best performing combinations were studied in more detail by comparing Avicel hydrolysis for several dilutions of the cell-free yeast culture supernatants. The dilutions displaying the most similar cellulose conversion rates are plotted in Additional file 3C to enable comparison between the samples. These data show that culture supernatant of *Sc*[*Tecbh1-TrCBM-C*] was two and a half times, *Sc*[*Tecbh1-TrCBM-C *&*C.l.cbh2b*] was over six times, and *Sc*[*H.g.cbh1 *&*C.l.cbh2b*] was four and a half times more efficient in cellulose conversion than the *T.e*.CBH1.

### Consequences of CBM fusion and co-expression two *cbh *genes on CBH production

In an attempt to explain why some CBHs are secreted at high concentration while others are not, and why co-expression of two enzymes alters CBH production levels relative to single enzyme production levels, we investigated relative differences in *cbh *mRNA levels, in copy number of the expression vector, and in secretion stress-induced responses in a set of eight strains including both high and low cellulase producers. *S. cerevisiae *expressing *T.r.cbh1, T.e.cbh1, Tecbh1-TrCBM-C, Tecbh1-TrCBM-C & T.r.cbh2, Tecbh1-TrCBM & C.l.cbh2b, T.r.cbh2, C.l.cbh2b *and the empty vector control strain were grown in YPD medium for three days and sampled daily for RNA isolation and enzyme activity measurements.

**Figure 5:**
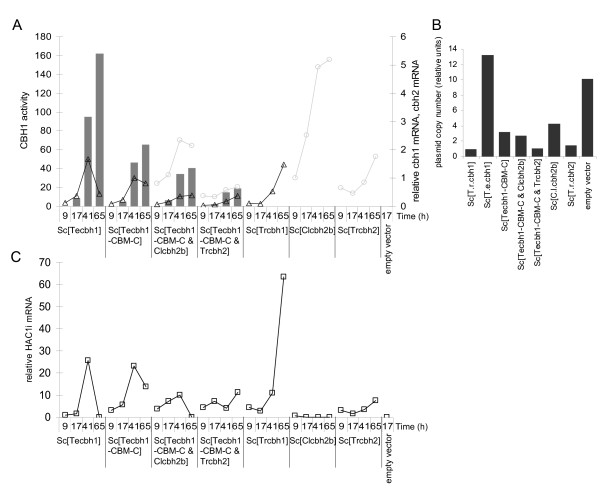
**Expression cellobiohydrolases and activation of the UPR**. Time course of CBH1 activity on MULac (grey bars), relative plasmid copy number (black bars), *cbh1 *mRNA (triangles), *cbh2 *mRNA (open circles) and *HAC1i *mRNA (open squares). **(a) **The *T.e.cbh1 *probe corresponding to the catalytic domain of *T.e*.CBH1, and the *T.r.CBM *probe, corresponding to the *T. reesei cbh1 *CBM were used for *cbh1 *mRNA detection on two identical Northern blots; hybridized separately with the two radioactively labeled probes that had the same specific activity. The signals were detected using a Typhoon scanner and quantified. The *cbh1 *hybridization signals were first normalized to *ACT1 *and then to *T.e.cbh1-CBM *signal at 41 hours. RNA was isolated after 9, 17, 41 and 65 hour cultivation, and enzyme activities on MULac in the culture supernatants were determined at 17, 41, and 65 hours. Quantification of *C.l.cbh2b *mRNA and *T.r.cbh2 *mRNA were done as explained above for *cbh1*. **(b) **Relative plasmid copy number (black bars) in yeast cells grown overnight in YPD. The hybridization signals were normalized to *T.r.cbh1 *signal set as 1. **(c) ***HAC1 *hybridization signal was first normalized to *ACT1 *and then to *T.e.cbh1 *signal at 9 hours and expressed as relative units.

Comparison between the different strains expressing *T.e.cbh1 *or its derivative with the *T. reesei *CBH1 CBM attached at the C-terminus showed that the highest steady state *T.e.cbh1 *mRNA levels and the highest enzymatic activity against MULac were produced by the *Sc*[*T.e.cbh1*] strain (catalytic domain only) followed by strains expressing *Sc*[*Tecbh1-TrCBM-C*], *Sc*[*Tecbh1-TrCBM-C & C.l.cbh2b*] and *Sc*[*Tecbh1-TrCBM-C & T.r.cbh2*] in this order throughout the cultivation (Figure 5A). Moreover, the *cbh1 *mRNA and enzyme activity levels in these strains also correlated positively with the plasmid copy number that was remarkably high in *Sc*[*T.e.cbh1*] (Figure 5A). In the strains co-expressing *Tecbh1-TrCBM-C *and either one of the two *cbh2 *genes, the mRNA levels of both *cbh1 *and *cbh2 *were decreased when compared with the corresponding strains expressing only one *cbh*, which is consistent with the plasmid copy numbers (Figure 5B).

Among the strains co-expressing *cbh1 *and *cbh2 *the copy numbers were relatively low, but interestingly there was a notable exception. The strain expressing *T.e.cbh1 & C.l.cbh2b*, the combination of the most highly expressed *cbh1 *and *cbh2 *genes, had a copy number intermediate to strains *Sc*[*T.e.cbh1*] and *Sc*[*C.l.cbh2b*] (Additional file 4A). Thus, irrespective of its larger size, the plasmid with two *cbh *expression cassettes, each about 3 kb, existed in more copies than a plasmid with only one expression cassette.

The expression of the unfolded protein response regulator *HAC1 *was studied as it is a sensitive indicator of UPR induction. The *HAC1^u ^*transcript that does not code for a functional protein was detected in all cells (Additional file 4B). The spliced *HAC1^i ^*mRNA coding for the UPR-inducible transcription factor was not detected in the strain containing the empty vector, while it appeared in each of the CBH strains, indicating that UPR was induced (Figure 5C and Additional file 4B). Expression of two other genes, *KAR2*(Bip) and *PDI1*, that are known to be induced by UPR, were also analyzed and their transcript levels were elevated relative to the empty vector control providing additional evidence for UPR in the cells (Additional file 4B).

The level of the *HAC1^i ^*mRNA varied between the strains so that the *Sc*[*C.l.CBH2b*] strain had the lowest *HAC1^i ^*levels at each time point throughout the cultivation suggesting that expression of this protein was the least stressful for the cell's secretion machinery. Similarly, the strain *Sc*[*T.e.cbh1*] producing an efficiently secreted enzyme also had relatively low levels of *HAC1^i^*. Comparison between the *Sc*[*T.e.cbh1*] and *Sc*[*Tecbh1-TrCBM-C*] strains showed that the strain expressing the bi-modular enzyme had 2-3 fold higher *HAC1^i ^*mRNA levels suggesting that production of the fusion protein caused a higher ER stress. Furthermore, the production of *T.r*.CBH1 protein caused a relatively strong UPR induction as judged by the *HAC1^i ^*mRNA level which suggests that the post-translational processing in the secretory pathway was impaired resulting in secretion of less than 1 mg/L of active *T.r*.CBH1 protein.

### CBH1 and CBH2 production in bioreactor

Strains M0759 expressing *Tecbh1-TrCBM-C *and M0969 expressing *C.l.cbh2b *(Tables 1 and 2), derived from the industrial background strain M0749 and disrupted in both copies of the *FUR1 *gene, were evaluated for their ability to accumulate CBH1 and CBH2 proteins during aerobic glucose fed-batch high cell density cultivation.

**Figure 6:**
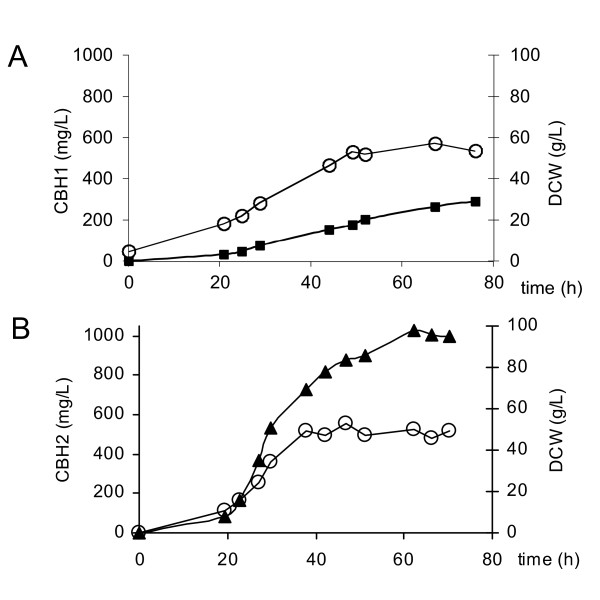
**Time course of protein accumulation during aerobic glucose fed-batch propagation of CBH producing strains in a 2L working volume bioreactor**. Accumulation of CBH1 (black squares), CBH2 (black triangles), and DCW (open circles) in culture media. **(a) **Strain M0759[*Tecbh1-TrCBM-C*]. **(b) **Strain M0969[*C.l.cbh2b*]).

The batch phase, which was defined by the end of ethanol consumption (see methods), took about 20 hours. Both strains reached the maximum dry cell weight (DCW) at about 40 hours of propagation but the CBH protein level continued to elevate for many hours after the DCW stopped increasing. Strain M0759, expressing *Tecbh1-TrCBM-C*, produced about 0.3 g/L of CBH (Figure 6A), and strain M0969, expressing *C.l.cbh2b*, accumulated about 1 g/L CBH (Figure 6B), determined by phenyl reversed-phase HPLC analysis. To our knowledge, this is the first demonstration of *S. cerevisiae *being able to accumulate exogenous CBH to such high titers, and to such high cell specific quantities. Considering that about half of yeast DCW consists of protein [34] we can estimate that *S. cerevisiae *is able to produce up to 4% of total cell protein as *C.l*.CBH2b.

The experiment was also performed for Y294 derived laboratory strains expressing *Tecbh1*-*TrCBM-C *or *C.l.cbh2b*. The laboratory strains reached three-fold less DCW at the end of glucose feed. Proportionally, the strains yielded three- to four-fold less protein per volume. Therefore, even though industrial strains were capable of reaching significantly higher biomass in aerobic bioreactor propagation conditions, DCW normalized protein production was similar for industrial and laboratory strains.

### Anaerobic Avicel fermentation with the aid of externally added β-glucosidase

As shown above, glucose accumulated during the incubation of cell-free culture supernatants of *Sc*[*cbh*] strains with Avicel cellulose and Novozyme 188 β-glucosidase *in vitro *(Figures 1, 2, 3, 4). In order to demonstrate the ability of the cellulolytic yeast to convert crystalline cellulose to soluble sugars, and further to ethanol, *in vivo *under typical yeast cultivation conditions, the following experiment was carried out. The strains expressing *Tecbh1-TrCBM-C *and *C.l.cbh2b *separately or in combination and the empty vector control were grown aerobically on YP-2% glucose medium for three days, and then Avicel cellulose was added to 20 g/L into the cultivation, and the incubation was continued under anaerobic conditions to prevent consumption of the ethanol that would be produced. Since the recombinant *S. cerevisiae *does not metabolize cello-oligosaccharides, Novozyme 188 β-glucosidase was added into the cultivations to enable conversion of cellobiose to glucose and subsequent fermentation, while parallel control flasks were not supplemented with Novozyme 188.

**Figure 7:**
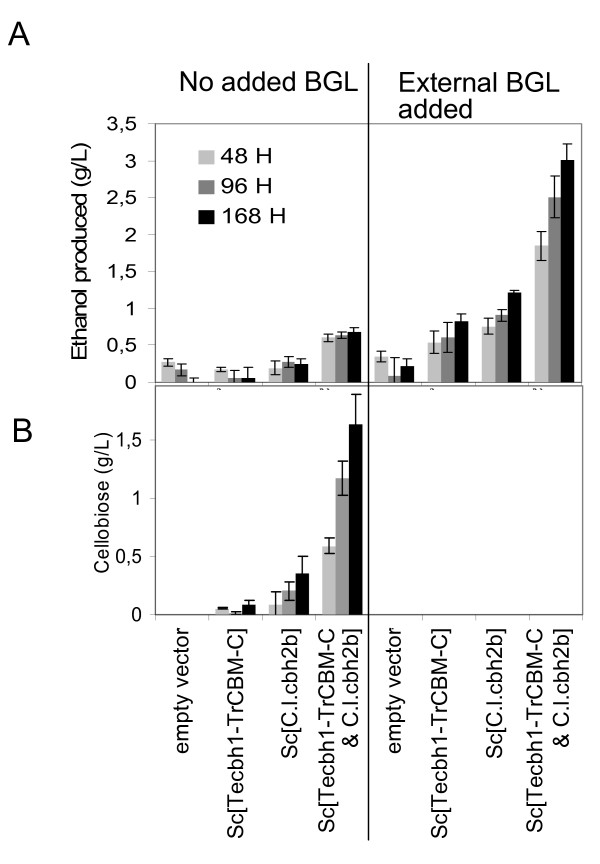
**Conversion of Avicel cellulose to ethanol with the aid of externally added BGL by *S. cerevisiae *secreting cellobiohydrolases**. **(a) **Levels of ethanol measured for the strains at 48, 96 and 168 hours. **(b) **Levels of accumulated cellobiose measured for the strains at 48, 96 and 168 hours. The values shown are the mean values of three repeats ± standard deviation.

The concentrations of ethanol, glucose and cellobiose were measured after 48, 96 and 168 hours of cultivation. These data show for the first time that the *S. cerevisiae*-produced CBH enzymes hydrolyzed crystalline cellulose to cello-oligosaccharides, which were further fermented to ethanol in the presence of externally added β-glucosidase (Figure 7). It should be noted that the rate of cellulose hydrolysis was high enough to allow sufficient glycolytic flux to enable fermentation. The concentration of ethanol increased over time for all the *Sc*[*cbh*] strains but not for the empty vector control strain, which proves the requirement of CBH for ethanol formation and shows that the components in the Novozyme 188 preparation are not sufficient for significant cellulose hydrolysis. The strain co-expressing *Tecbh1-TrCBM-C *and *C.l.cbh2b *that converted approximately 23% of the Avicel to soluble sugars *in vitro *produced up to 3 g/L ethanol from 20 g/L cellulose, corresponding to approximately 30% of theoretical maximum yield during the cultivation conditions when supplemented with Novozyme 188. In the case where Novozyme 188 was not added, cellobiose accumulated in the medium up to 1.6 g/L.

## Discussion

High level functional expression and secretion of exoglucanases are requirements for enabling non-cellulolytic organisms such as *S. cerevisiae *to utilize crystalline cellulose substrates [35]. The difficulty of producing CBHs in sufficient quantities is considered as a major hurdle in the development of yeast as a CBP organism [6,19,36]. In this study we have attempted to alleviate this problem through identifying gene candidates that are compatible with expression in yeast. To this end we screened 14 *cbh1 *(Cel7A) and 10 *cbh2 *(Cel6A) encoding genes from ascomycetous origin by functional expression in *S. cerevisiae*. Somewhat surprisingly, despite sequence conservation and similar folding within CBH1s and also within CBH2s from different species, a wide range of enzyme production levels was observed even though the same regulatory sequences for all the *cbh1 *genes (*ENO1 *promoter and terminator) and *cbh2 *genes (*PGK1 *promoter and terminator), the same vector backbone and the same host cells were used. The *T.r*.CBH1 included in our study for comparison was produced at levels comparable to earlier reports of 0.2-5.0 mg/L of *T.r*.CBH1 [15,19,25], confirming the disappointingly poor production. However, we identified several other *cbh1 *genes that were expressed more efficiently: the activity of *T.e*.CBH1 and its derivative with the *T.r*.CBM attached to its C-terminus exceeded that of *T.r*.CBH1 by at least two orders of magnitude, yielding 100-200 mg/L in shake flasks and 300 mg/L in high cell density conditions. This shows a large improvement compared with a recent report of 5-10 mg/L *T.e*.CBH1 [24]. Heinzelman *et al*. [24] expressed *cbh1 *genes of *T. reesei, T. emersonii, A. thermophilum, C. thermophilum *and *T. aurantiacus*. The activity based ranking is similar to our results, with the exception of *T. aurantiacus*, however the enzyme secretion levels were higher in our work, which may result from differences in codon optimization, or strain and construct specific effects or, in the case of *T. aurantiacus*, a difference in the amino acid sequences chosen initially. The highest CBH level secreted, 1000 mg/L *C.l*.CBH2b, corresponding to 4% of the total cellular protein, was produced in high cell density conditions, exceeded any previous reports on CBH production in *S. cerevisiae*. In the shake flask cultivations on defined medium the difference between secreted *C.l*.CBH2b and *T.r*.CBH2 was two- to three-fold, the *T.r*.CBH2 level being comparable with earlier reported levels of 10-100 mg/L [15,16]. In contrast to earlier conclusions, the present work proves that *S. cerevisiae *is capable of secreting CBHs at high levels that compare well with the highest heterologous protein production levels described for *S. cerevisiae *[27,37,38].

The differences in secreted enzyme levels can be explained to a large extent by the differences in plasmid copy number, which were highest for the strains secreting the highest enzyme levels, *Sc*[*T.e.cbh1*], *Sc*[*C.l.cbh2b*], and *Sc*[*Tecbh1-TrCBM-C *&*C.l.cbh2b*]. While the copy number and segregation of the endogenous 2-micron circle is under strict control [39], little is known about copy number control of artificial 2-micron plasmids [40,41] even though they are widely used essential molecular biology tools, and the best option to ensure high expression level, which is necessary for addressing secretability of the proteins of interest. The suggestion that the plasmid size may affect its copy number and stability [40] seems unlikely in our case since the sizes differed by less than 1% within the *cbh1 *expressing plasmids and within the *cbh1 *and *cbh2 *co-expressing plasmids. It appears that the *cbh *gene inserts influence the plasmid copy number significantly, but the mechanism by which this occurs remains unknown. Possibly the *cbh *inserts affect plasmid replication or transcription, or indirect cellular effects caused by the *cbh *translation products may be involved.

Among the strains expressing *T.e.cbh1 *or its derivatives, the plasmid copy number, CBH1 enzyme activity and *cbh1 *mRNA levels were consistent, all of which were the highest for the strain *Sc*[*T.e.cbh1*] followed by strains expressing *Sc*[*Tecbh1-TrCBM-C*], *Sc*[*Tecbh1-TrCBM-C *&*C.l.cbh2b*] and *Sc*[*Tecbh1-TrCBM-C *&*T.r.cbh2*] in that order. With regard to the last two strains, it appeared that the presence of *C.l.cbh2b *allowed higher plasmid, mRNA and activity levels than *T.r.cbh2 *did, similarly to strains *Sc*[*C.l.cbh2b*] and *Sc*[*T.r.cbh2*]. Moreover, it appears that the attachment of the linker and CBM moieties to the *T.e*.CBH1 catalytic domain decreased the plasmid copy number, transcript and enzyme levels. The results indicate that individual gene and/or protein specific features and compatibility with the host are important, not only for efficient production of the individual protein but also for efficient production of the accompanying protein, when two or more genes are simultaneously expressed from one plasmid. From this point of view, expression of integrated gene copies could be a useful strategy; however, high level expression is likely to require integration of each gene in multiple copies. Multicopy integration has also been applied in the construction of yeast strains expressing cellulases [42,43].

The *T.r.cbh1 *mRNA level and plasmid copy number were comparable to those of strain *Sc*[*Tecbh1-TrCBM-C *&*T.r.cbh2*] and therefore it could be expected that a reasonable amount of *T.r*.CBH1 activity would have been detected. Because activity was barely detectable, it implies that post-transcriptional events have a major impact on the production and secretion of this enzyme from *S. cerevisiae*. It is possible that the activity of *Tr*CBH1 was impaired due to hyperglycosylation or incorrect folding to some extent, as has been suggested previously [15,25,26].

The secretion of heterologous proteins is believed to be limited by processes occurring in the ER [32,44]. The machinery required for proper protein folding may become saturated when heterologous proteins are over expressed, causing accumulation of misfolded or aggregated proteins in the ER. The UPR regulates gene expression in response to ER stress, resulting in selective induction of genes that are essential under folding stress and in specific remodeling of the secretory pathway to improve the protein folding capacity [32]. The transcription factor Hac1p is the central regulator mediating the UPR [30,31]. It has been suggested before that some components of the UPR pathway are involved in the secretion of *T. reesei *endoglucanase, which was based on a comparison between two *S. cerevisiae *strains, one with an intact *HAC1 *gene and the other with a disrupted *hac1 *gene preventing the UPR [45]. Since the effects of cellulase production on the secretory machinery have not been studied before in *S. cerevisiae*, we undertook northern analysis of the UPR-related genes to provide insight into the physiological responses associated with CBH production. Especially *HAC1^i^*, but also *PDI1*, and *KAR2 *mRNA levels were elevated in the strains expressing certain *cbh1 *and/or *cbh2 *genes relative to the empty vector control, which showed that the expression of the CBHs studied caused ER stress and activated the UPR in the cells in order to adapt to the prevailing conditions. This is to our knowledge the first demonstration of UPR activation in *S. cerevisiae *in response to cellulase expression.

The strength of UPR activation varied depending on the gene as judged from the expression levels of the *HAC1^i ^*mRNA. Interestingly, a negative correlation between the *HAC1^i ^*mRNA levels and the amount of secreted active enzyme was found. For example, the *Sc*[*C.l.cbh2b*] strain which secreted the highest amount of CBH had the lowest *HAC1^i ^*mRNA level of all the *cbh*-expressing strains studied. This suggests that the protein folding capacity was greater in strain *Sc*[*C.l.cbh2b*] than for the other strains, which enabled high level secretion of *C.l*.CBH2b. The *Sc*[*Tecbh1-TrCBM-C*] also had a relatively high *HAC1^i ^*mRNA level and low enzyme level compared with the strain *Sc*[*T.e.cbh1*], which suggests that additional ER protein folding capacity was required to produce the bimodular enzyme, in which the formation of two additional disulfide bridges is necessary for correct folding of the CBM. Furthermore, it appears that the *T.r*.CBH1 and *T.r*.CBH2 proteins were more potent than, for example, *T.e*.CBH1 or *C.l*.CBH2b in causing secretion stress and inducing the UPR, even though the gene copy number and resultant expression level of *T.r.cbh1 *and *T.r.cbh2 *were lower than those of the abundantly expressed *T.e.cbh1 *or *C.l.cbh*2b. It may be possible that, despite the strong UPR, the ER protein folding capacity was not sufficient to enable efficient secretion of, for example, *T.r*.CBH1.

## Conclusions

As concluded above, the plasmid copy number could explain secreted CBH levels to a large extent. At the same time, the expression of certain genes induced a stress response in the ER and upregulation of the UPR correlated with low plasmid copy number. It would appear that some CBHs are thus more compatible with high-level expression and production in *S. cerevisiae *than others, although which features lead to incompatibility, marked by low levels of plasmid, mRNA and secreted protein and strong induction of UPR, are difficult to define. The stress response indicated that CBH production was a burden to the cells. One way to relieve the stress could be downregulation of CBH production either through UPR or through decreasing the plasmid copy number. Whether there is a link between ER stress and plasmid copy number control or whether they occur independently of each other cannot be concluded from our data, but would require a separate study.

*S. cerevisiae*, the most efficient ethanol producer on a large industrial scale, was shown to be capable of high level CBH expression. This indicates that it is a promising organism for conversion of cellulosic biomass to ethanol. The main obstacle in the way of applying CBP with *S. cerevisiae *is considered to be the sufficiency of CBH production, estimated to require approximately 20- to 120-fold improvement [19]. The progress made in the present work demonstrated that production of both CBH1 and CBH2 could be improved to that level and that the barrier of CBH sufficiency was overcome. Data demonstrating that yeast co-expressing CBH1 and CBH2 could ferment Avicel cellulose to ethanol with the aid of externally added β-glucosidase supports this conclusion. Simultaneous expression of CBHs with endoglucanases and β-glucosidase is the next step to enable *S. cerevisiae *to directly convert cellulose to ethanol and to grow on cellulose under CBP conditions. It can be envisioned that cellulolytic *S. cerevisiae *will also be suitable for other biorefinery process concepts, exploiting the capability of yeast to convert cellulose to useful products other than ethanol.

## Methods

### Strains, media and culture conditions

*Escherichia coli *strains XL1 Blue MRF' (Stratagene, La Jolla, CA, USA) and DH5α were used for cloning. *S. cerevisiae *Y294 (α*leu2*-*3,112 ura3-52 his3 trp1-289*) [ATCC 201160] was used as the host for CBH expression. *S. cerevisiae *M0749 (Mascoma proprietary industrial strain) [46] was used as the host for larger scale production for CBH1 and CBH2 enzyme purification. Yeast were grown at 30°C with shaking in YPD and SCD media supplemented with the necessary amino acids as required (Additional file 5).

### Plasmid and strain construction

Standard DNA techniques [47] were used in the study. Details about plasmids and recombinant strains used and constructed are summarized in Tables 1, 2 and 3. The nucleotide sequences of the *cbh *genes were codon-optimized for expression in *S. cerevisiae *and synthesized by *de novo *gene synthesis providers (Table 1). Synthetic genes were subsequently cloned onto yeast expression vectors containing the *URA3 *selection marker and the 2-micron sequence for autonomous replication. The *cbh1 *genes were expressed under transcriptional control of the *S. cerevisiae *enolase gene (*ENO1*) promoter, and the *cbh2 *genes under the *S. cerevisiae *3-phosphoglycerate kinase gene (*PGK1*) promoter and terminator. To attach carbohydrate-binding modules (CBMs) to the CBM-less *T.e*.CBH1, PCR was used (Table 2). For simultaneous expression of *cbh1 *and *cbh2 *genes the *ENO1*p*-cbh1-ENO1*t and the *PGK1*p*-cbh2-PGK1*t expression cassettes were cloned into the same vector in different combinations (Table 3). *S. cerevisiae *was transformed with the lithium acetate dimethylsulfoxide method [48] and selected for uracil prototrophy on SCD^-URA^. Autoselective strains were constructed to ensure maintenance of the *URA3*-bearing expression vectors in complex medium (Additional file 5).

### Enzyme assays

To determine exoglucanase activity on a polymeric insoluble substrate, 300 μL of the yeast culture supernatant was added to deep-well microtiter plates with each well containing 300 μL of 2% Avicel PH-105 cellulose (FMC Biopolymer, Mechanicsburg, PA, USA), 0.05 M acetate buffer pH 5.0, 0.04% sodium azide and 0.3 μL Novozyme-188 (Sigma-Aldrich, St Louis, MO, USA) at approximately 1000 rpm at 35°C. The amount of sugars released during 24 h and 28 h incubations was determined using a modified 3,5-dinitrosalicylic acid (DNS) method (Additional file 5). Glucose was used to set a standard curve in the range of 0.125 to 4 g/L from which the amount of glucose released during the assay was determined. The amount of activity was expressed as the percentage of Avicel hydrolyzed.

Secreted activities for the strains producing CBH1 (GH7) enzymes were determined using soluble 4-methylumbelliferyl-β-D-lactoside (MULac, Sigma) (Additional file 5).

### Protein purification

*Te*CBH1*-Tr*CBM-C and *C.l*.CBH2b were purified using chromatography methods for use as protein standards in the HPLC assay (Additional file 5).

### Other protein analysis methods

Protein concentrations in shake flask cultivations were measured with BioRad protein reagent (Additional file 5). Endoglycosidase H (Roche, Mannheim, Germany) was used to remove *N*-linked glycans (Additional file 5). Protein samples were separated on SDS-PAGE gels (BioRad) and visualized with silver staining.

For determination of the concentration of CBHs produced in bioreactor cultivations, a phenyl reversed phase method was developed on HPLC, and the purified *Te*CBH1*-Tr*CBM-C and *C.l.CBH2b *were used for generating a standard curve (Additional file 5).

### Determination of plasmid copy number

Yeast DNA was isolated by phenol extraction from cells grown overnight in YPD (Additional file 5). Radioactive *URA3 *hybridization signals on Southern blots were quantified as described in Additional file 5 and the plasmid copy number was determined as the ratio between the plasmid-borne and the genomic copy of *URA3*. The copy number is expressed in relative units.

### Gene expression studies

Yeast were grown in 50 mL YPD medium in 250 mL Erlenmeyer flasks at 30°C at 250 rpm and 2 mL samples were removed periodically. Cells were harvested by centrifugation, frozen in dry ice and stored at -70°C. RNA was isolated using the Trizol reagent (Invitrogen cat. no. 15596-018). Northern blots were prepared and hybridized with *T.e.cbh1, T.r.cbh2, C.l.cbh2b, T.r.cbh1 CBM, HAC1, ACT1, KAR2 *and *PDI1 *probes (Additional file 6) using conventional techniques [47]. Radioactive hybridization signals were detected and quantified as above.

### Bioreactor propagation of CBH-producing yeast strains

Aerobic glucose fed-batch high cell density cultivation was performed in 2 liters working volume bioreactors with strains M0759 and M0969 (Additional file 5).

### Avicel fermentation to ethanol

The yeast strains were grown in YPD medium for four days (Additional file 5). Subsequently, 25 mL of each culture was added to McCartney bottles containing 0.5 g of Avicel PH-105 to attain a concentration of 20 g/L. In addition, 100 μL of the β-glucosidase preparation Novozyme 188 (Sigma) was added so that for each strain there were triplicate bottles with and without added enzyme. The bottles were sealed with rubber lined caps to maintain the cultures anaerobically and stirred on magnetic stirrers for seven days. Samples were taken on days 0, 2, 4 and 7 and cellobiose, glucose and ethanol content was determined with HPLC (Additional file 5).

## Abbreviations

CBH: cellobiohydrolase enzyme; CBM: carbohydrate-binding module; CBP: consolidated bioprocessing; DCW: dry cell weight; DNS: 3,5-dinitrosalicylic acid; ER: endoplasmic reticulum; HPLC: high performance liquid chromatography; MULac:4-methylumbelliferyl β-D-lactoside; PCR: polymerase chain reaction; SCD: synthetic complete dextrose medium; SSF: simultaneous saccharification and fermentation; UPR: unfolded protein response; YPD: yeast extract peptone dextrose.

## Competing interests

Some of the work presented in this paper was also filed in international patent application WO/2010/060056.

## Authors' contributions

MI, RDH, EB, JMB, AF, AK, SPV, MS, VR, WHVZ and MP designed research; MI, RDH, EB, EW, AF, SPV, DLG, MS, SA, KD, and MM performed research; MI, RDH, EB, JMB, EW, AF, SPV, DLG, MS, VR analyzed data; MI, RDH, EB, JMB, EW, AF, SPV, and WHVZ, and MP wrote and edited the manuscript; EB, JMB, EW, NT contributed new analytic tools. All authors read and approved the final manuscript.

## Supplementary Material

Additional file 1 **Secreted CBH1 activity**. This figure shows the secreted MULac activity produced by recombinant strains expressing *cbh1 *genes cultured in YPD and in SCD media, and Avicel hydrolysis by the supernatants of the same strains. Click here for file

Additional file 2 **Partial amino acid sequence alignment of the C-terminal end of *T.e*.CBH1 fused to the *T. reesei, C. thermophilum *or *H. grisea *linker-CBM sequences**. Click here for file

Additional file 3 **Secreted CBH activity produced by recombinant strains co-expressing *cbh1 *and *cbh2 *genes**. This figure shows the secreted MULac activity produced in YPD medium by recombinant strains co-expressing *cbh1 *and *cbh2 *genes in 10 different combinations together with strains expressing the single *cbh *genes, and Avicel hydrolysis by the supernatants of the same strains. In addition, Avicel hydrolysis by the best performing cell-free yeast culture supernatants in several dilutions is shown. Click here for file

Additional file 4 **Plasmid copy number and UPR-related mRNAs in strains expressing *cbh1 *and/or *cbh2 *genes**. This figure shows the relative plasmid copy number in 17 *S. cerevisiae *strains in panel A. In panel B, the results of Northern analyses and quantification of *HAC1, KAR2 *and *PDI1 *mRNAs are shown. Click here for file

Additional file 5 **Methods**. This file provides a detailed description of the methods used. Click here for file

Additional file 6 **Oligonucleotides and restriction fragments used for preparation of probes**. This table identifies the nucleotide sequences of the probes used in the Northern hybridizations. Click here for file
