# Supplementary material for: High level secretion of cellobiohydrolases by Saccharomyces cerevisiae
Source: Biotechnol Biofuels. 2011 Sep 12;4:30. doi: 10.1186/1754-6834-4-30 (PMC3224389; doi:10.1186/1754-6834-4-30)
Supplement: Additional file 1 — Secreted CBH1 activity. This figure shows the secreted MULac activity produced by recombinant strains expressing cbh1 genes cultured in YPD and in SCD media, and Avicel hydrolysis by the supernatants of the same strains. [file 1754-6834-4-30-S1.PDF]

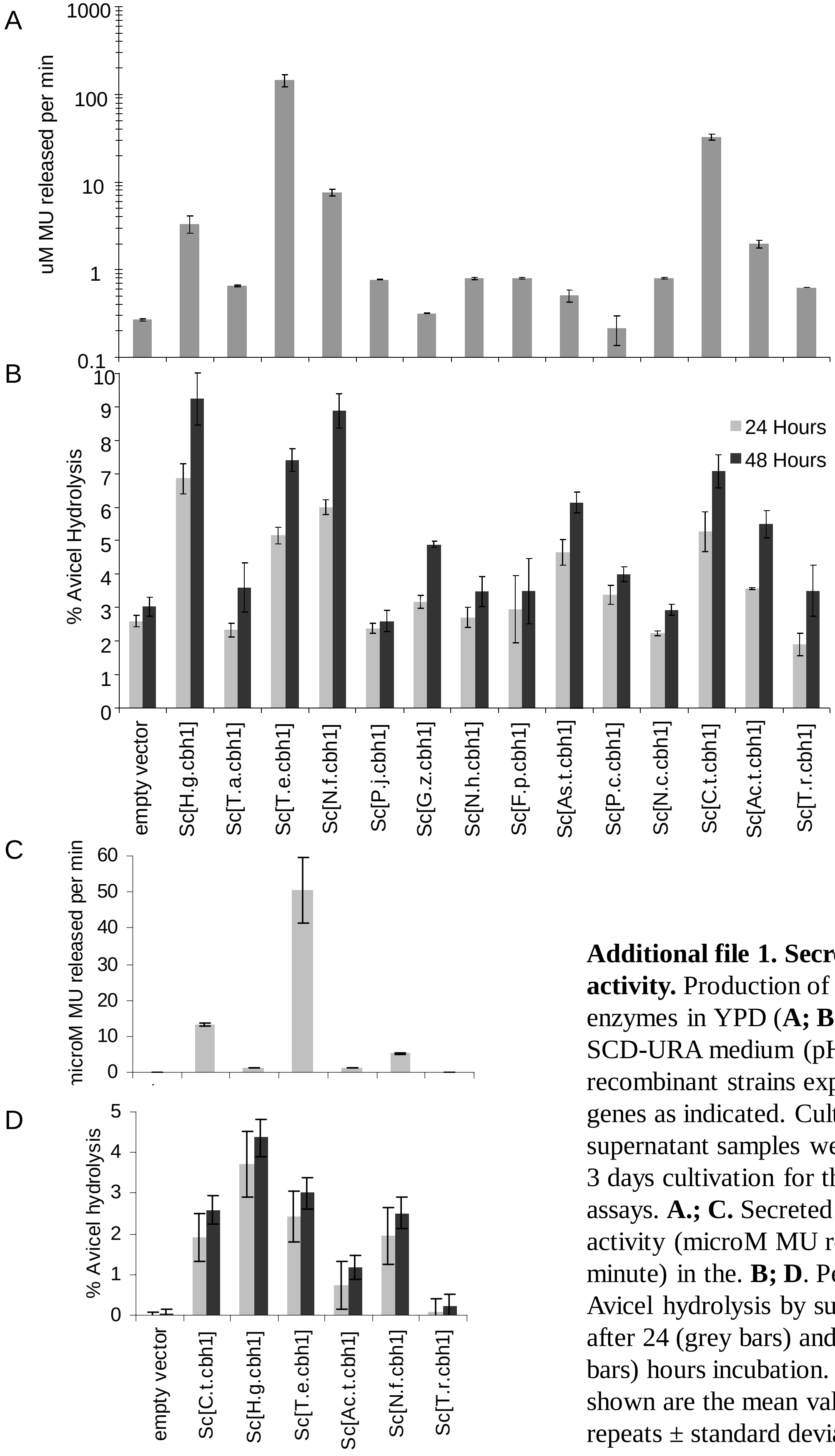

Additional file 1. Secreted CBH1 activity. Production of CBH1 enzymes in YPD (**A; B**) and in SCD-URA medium (pH 6) (**C**; **D**) by recombinant strains expressing *cbh1* genes as indicated. Culture supernatant samples were taken after 3 days cultivation for the enzyme assays. **A.; C.** Secreted MULac activity (microM MU released per minute) in the. **B; D**. Percentage Avicel hydrolysis by supernatants after 24 (grey bars) and 48 (black bars) hours incubation. The values shown are the mean values of three repeats ± standard deviation (SD).
