# Supplementary material for: High level secretion of cellobiohydrolases by Saccharomyces cerevisiae
Source: Biotechnol Biofuels. 2011 Sep 12;4:30. doi: 10.1186/1754-6834-4-30 (PMC3224389; doi:10.1186/1754-6834-4-30)
Supplement: Additional file 2 — Partial amino acid sequence alignment of the C-terminal end of T.e.CBH1 fused to the T. reesei, C. thermophilum or H. grisea linker-CBM sequences. [file 1754-6834-4-30-S2.PDF]

```
Tecbh1-TrCBM-C  420 VPSDVESQSPNSYVTYSNIKFGPINSTFT---ASNP------------PGGN 461
Tecbh1-CtCBM-C  420 VPSDVESQSPNSYVTYSNIKFGPIGSTVPGLDGSNPGNPTTTVVPPASTSTS 476
Tecbh1-HgCBM-C  420 VPSDVESQSPNSYVTYSNIKFGPIGSTVAGLPGAGNGGNN-------GGNPP 469
                    ************************.**..   .:.               .

Tecbh1-TrCBM-C      RGTTTTRRPATT-TGSSPGPTQSHYGQCGGIGYSGPTVCASGTTCQVLNPYYSQCL 516
Tecbh1-CtCBM-C      RPTSSTSSPVSTPTGQPGGCTTQKWGQCGGIGYTGCTNCVAGTTCTQLNPWYSQCL 532
TeCBH1-HgCBM-C      PPTTTTSSAPATTTTASAGPKAGRWQQCGGIGFTGPTQCEEPYICTKLNDWYSQCL 525
                      *::*  . :* *  . * .  :: ******::* * *     *  ** :*****
```

Additional file 2. Partial amino acid sequence alignment of the C-terminal end of *T.e.*CBH1 fused to the *T. reesei, C. thermophilum* or *H. grisea* linker-CBM sequences. The last amino acid of the native *T.e.*CBH1, Ser455, is shown in **bold** type in the *Te*CBH1-CBM-C sequence. The cysteine residues in the CBM that take part in disulfide bridge formation are shown in **bold** type and the aromatic amino acids predicted to bind cellulose are underlined. The *T.e.*CBH1-*T.r.*CBM-C enzyme has an additional N-glycosylation target site (Asn449) lacking from the other two bi-modular enzymes.
