# Supplementary material for: High level secretion of cellobiohydrolases by Saccharomyces cerevisiae
Source: Biotechnol Biofuels. 2011 Sep 12;4:30. doi: 10.1186/1754-6834-4-30 (PMC3224389; doi:10.1186/1754-6834-4-30)
Supplement: Additional file 3 — Secreted CBH activity produced by recombinant strains co-expressing cbh1 and cbh2 genes. This figure shows the secreted MULac activity produced in YPD medium by recombinant strains co-expressing cbh1 and cbh2 genes in 10 different combinations together with strains expressing the single cbh genes, and Avicel hydrolysis by the supernatants of the same strains. In addition, Avicel hydrolysis by the best performing cell-free yeast culture supernatants in several dilutions is shown. [file 1754-6834-4-30-S3.PDF]

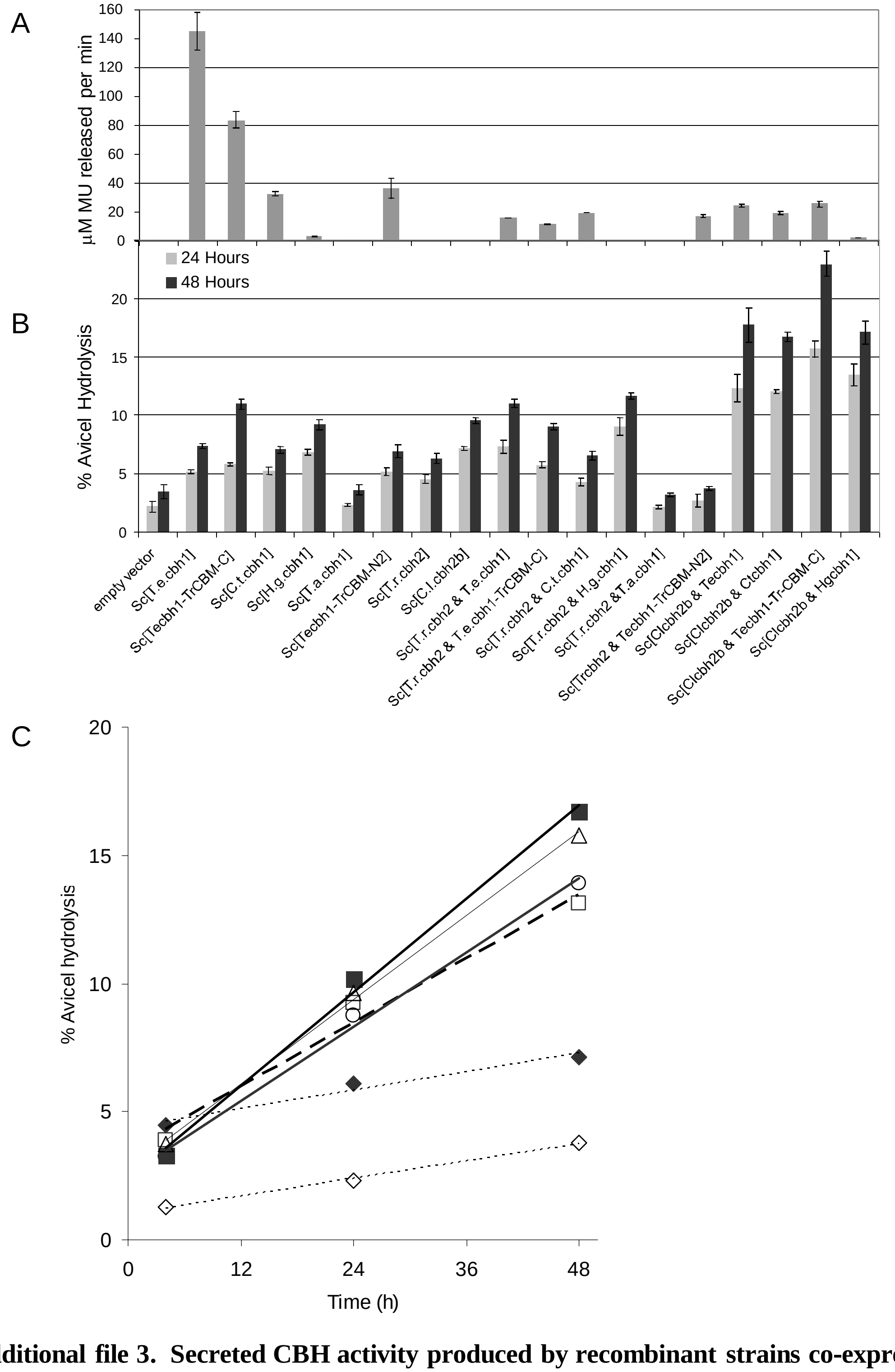

Additional file 3. Secreted CBH activity produced by recombinant strains co-expressing *cbh1* and *cbh2* genes. The strains expressing the corresponding single *cbh1* and *cbh2* genes are included for comparison. Activities in cell-free culture supernatants were measured after three days cultivation in YPD medium. **A.** Secreted MULac activity (microM MU released per minute) and **B**. Percentage of avicel hydrolysis by supernatants of the same strains in 24 and 48 hours. The values shown are the mean values of three repeats ± SD. **C.** Avicel hydrolysis by diluted cell-free culture supernatants of yeast strains *Sc*[*Tecbh1-CBM & Clcbh2b*] diluted 1:12 (■), *Sc*[*Hgcbh1 & Clcbh2b*] diluted 1:9 (○), *Sc*[*T.e.cbh1*] diluted 1:2 (□), *Sc*[*T.e.cbh1-TrCBM-C*] diluted 1:5 (Δ), and *Sc*[empty vector] non-diluted (♦). The control sample containing buffer (◊) instead of culture supernatant is also shown. The reaction mixture was sampled after 4, 24 and 48 h of incubation.
