# Supplementary material for: High level secretion of cellobiohydrolases by Saccharomyces cerevisiae
Source: Biotechnol Biofuels. 2011 Sep 12;4:30. doi: 10.1186/1754-6834-4-30 (PMC3224389; doi:10.1186/1754-6834-4-30)
Supplement: Additional file 4 — Plasmid copy number and UPR-related mRNAs in strains expressing cbh1 and/or cbh2 genes. This figure shows the relative plasmid copy number in 17 S. cerevisiae strains in panel A. In panel B, the results of Northern analyses and quantification of HAC1, KAR2 and PDI1 mRNAs are shown. [file 1754-6834-4-30-S4.PDF]

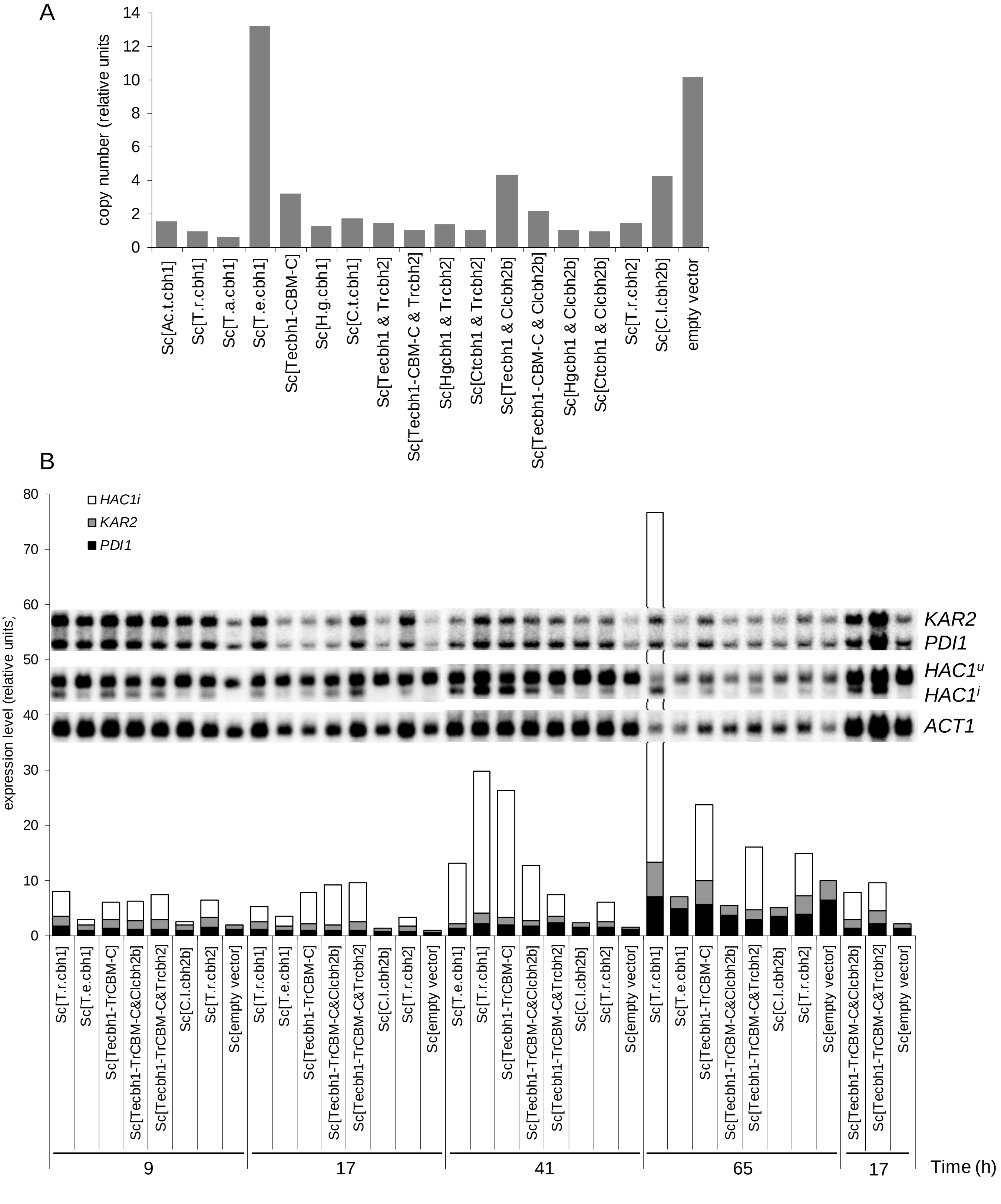

Additional file 4. Plasmid copy number and UPR related mRNAs in strains expressing *cbh1* and/or *cbh2* genes. A. Relative plasmid copy number in the *S. cerevisiae* strains indicated. The values were normalized to [*T.r.cbh1*] set as 1. The yeast were grown overnight in YPD for the determination of relative plasmid copy number. **B.** Northern analysis and quantification of *HAC1*, *KAR2*, *PDI1* mRNAs. Each hybridization signal was first normalized to *ACT1* and then to *T.e.cbh1* signal at 9h and expressed as relative units.
