# Supplementary material for: High level secretion of cellobiohydrolases by Saccharomyces cerevisiae
Source: Biotechnol Biofuels. 2011 Sep 12;4:30. doi: 10.1186/1754-6834-4-30 (PMC3224389; doi:10.1186/1754-6834-4-30)
Supplement: Additional file 5 — Methods. This file provides a detailed description of the methods used. [file 1754-6834-4-30-S5.DOC]

**Additional file 5.**

**Methods**

**Strains, media and culture conditions**

*Escherichia coli* strains XL1 Blue MRF’ (Stratagene; Δ(*mcrA*)*183* Δ(*mcrCB-hsdSMR-mrr*)*173 endA1 supE44 thi-1 recA1 gyrA96 relA1 lac*[*F*’ *proAB lacI*q*Z*Δ*M15 Tn10* (*Tet*r)]) and DH5α [*F-φ80dlacZΔM15Δ(lacZYA-argF)U169 recA1 endA1 tonA hsdR17(r_K-_, m_K+_) phoA supE44 λ -thi-1 gyrA96 relA1*] were used for plasmid transformation and propagation. Cells were grown in LB medium (5 g/liter yeast extract, 10 g/liter NaCl, 10 g/liter tryptone) supplemented with ampicillin (100 mg/liter). *S. cerevisiae* Y294 (α*leu2*–*3*,*112 ura3-52 his3 trp1-289*) [ATCC 201160] was used as the host for CBH expression. *S. cerevisiae* Y294 transformants were selected and maintained on SCD^-URA^ or SCD^-URA-LEU^ medium plates (1.7 g/liter yeast nitrogen base w/o amino acids and ammonium sulphate [Difco Laboratories, Detroit, MI, USA], 5 g/liter (NH_4_)_2_SO_4_, 20 g/liter glucose, 15 g/liter agar, and supplemented with amino acids as required). Autoselective *S. cerevisiae* strains were cultured in YPD medium (10 g/liter yeast extract, 20 g/liter peptone, 20 g/liter glucose). *S. cerevisiae* M0749 (Mascoma proprietary industrial strain) transformants were used as the host for larger scale production for CBH1 and CBH2 enzyme purification. Yeast strains were routinely cultured in 250 ml Erlenmeyer flasks containing 50 ml medium at 30 °C, on a rotary shaker at 150 rpm, unless otherwise stated. Recombinant enzyme production was carried out in 125 ml Erlenmeyer flasks containing 10 ml medium after strains were grown at 30 °C for 3 days.

**Plasmid and strain construction**

Standard DNA techniques [1] were used in the study. Enzymes for DNA modifications were purchased from Roche Diagnostics GmbH (Mannheim, Germany), New England Biolabs (Beverly, MA, USA), Fermentas (Vilnius, Lithuania) and Finnzymes (Espoo, Finland). PCR amplifications were performed using a Mastercycler® gradient (Eppendorf AG, Hamburg, Germany) or a Perkin Elmer GeneAmps PCR System 2400 (The Perkin–Elmer Corporation, Norwalk, CT, USA), and sequencing reactions were analyzed using an ABI Prism^TM^ 3100 Genetic Analyzer (Applied Biosystems, Foster City, CA, USA). Qiagen GmbH (Hilden, Germany) or Zymo Research (Orange, CA, USA) products were used for plasmid isolations and gel extractions. Details about plasmids and strains used and constructed in this study are summarised in Tables 1, 2, and 3.

The nucleotide sequences of the *cbh* genes expressed in this study were codon-optimized for expression in *S. cerevisiae* using the Codon Adaptation Index (CAI) calculator for expression in *S. cerevisiae* [2] or by proprietary gene design software from Geneart or Codon Devices. Sequences were based on amino acid sequences from Genbank (see Table 1 for accession numbers). Unwanted sequence repeats, polyadenylation sites, splice sites, potential secondary RNA structures and unwanted restriction endonucleases sites were avoided while optimal GC content and codon usage were prioritized. Native enzyme secretion signals were used unless otherwise shown (Table 1). The complete gene sequences including 5’ *Pac*I or *Eco*RI and 3’ *Asc*I or *Xho*I restriction endonuclease sites were sent for *de novo* gene synthesis to GenScript Corporation (Piscataway, NJ, USA), Geneart (Regensburg, Germany) or Codon Devices (Cambridge, MA, USA). Synthetic genes were excised from the plasmids that were provided by digesting with *Eco*RI and *Xho*I or with *Asc*I and *Pac*I and cloned onto yeast expression vectors containing the *URA3* selection marker and the 2-micron sequence for autonomous replication.

For expression of genes under transcriptional control of the *S. cerevisiae* enolase gene (*ENO1*) promoter and terminator (Additional files 1 and 3), genes were cloned as *Eco*RI/*Xho*I fragments into the plasmid yENO1 [3] or as *Pac*I/*Asc*I fragments into a derivative of yENO1 where a linker sequence containing the *Pac*I and *Asc*I sites was inserted between the promoter and terminator sequences. For expression of genes under transcriptional control of the *S. cerevisiae* 3-phosphoglycerate kinase gene (*PGK1*) promoter and terminator (Tables 1 and 2), genes were cloned as *Eco*RI/*Xho*I fragments into the plasmid pJC1 [4] or as *Pac*I/*Asc*I fragments into a derivative of pJC1 where a linker sequence containing the *Pac*I and *Asc*I sites was inserted between the promoter and terminator sequences.

To attach carbohydrate binding modules (CBMs) to the *T. emersonii cbh1*, overlap PCR was used. Details of the primers used are given in Table 2. To create the gene *Tecbh1-TrCBM-N2* (Table 2) the primers NCBM-L and NCBM-OL2 were used to amplify the *T. reesei xyn2* secretion signal – *T. reesei cbh2* N-terminal CBM fusion from the plasmid pRDH107. This 382 bp fragment was then used along with primer NCBM-R to amplify the *T. emersonii cbh1* from the plasmid pRDH105. The resulting 1695 bp fusion gene (*Tecbh1-TrCBM-N2*) was cloned into yENO1. To create the gene *Tecbh1-TrCBM-C2* (Table 2) the primers CCBM-L and CCBM-OL2n were used to amplify the *T. reesei xyn2* secretion signal – *T. emersonii cbh1* fusion from a plasmid pDLG116 (where native *T. emersonii* secretion signal was removed and replaced by the *T. reesei xyn2* secretion signal). This 1437 bp fragment was then used along with primer CCBM-R to amplify the *T. emersonii cbh1* catalytic domain from the plasmid pRDH105. The resulting 1611 bp fusion gene (*Tecbh1-TrCBM-C2*) was cloned into yENO1. To create the gene *Tecbh1-TrCBM-N* the primers 406TemCBH1 NCBM-F (that contains the *T.e.cbh1* secretion signal) and 407TemCBH1 NCBM-R were used to amplify the *T. reesei cbh2* CBM domain from pDLG117. The PCR product was cut with *Eco*RI and *Pml*I and ligated with *Eco*RI-*Pml*I cut pRDH105 producing pMI528. To create the *Tecbh1-TrCBM-*C gene the primers 395Tecbh1-F and 398Tecbh1-R were used to amplify *T.e.cbh1* from pRDH105, the PCR product was digested with *Pml*I and *Sma*I and the 800 bp fragment was isolated. The primers 399Trcbh1-F and 400Trcbh1-R were used to amplify the CBM from pRDH101, digested with *Mly*I and *Xho*I and the 180 bp fragment was isolated. The fragments were ligated with the 6.9 kb *Pml*I-*Xho*I fragment of pRDH105 resulting in pMI529. To construct the *Tecbh1-CtCBM-C* gene the primers 392ENO1p-F and 393TeCt-R were used to amplify the *ENO1*p*-T.e.cbh1,* the fragment was digested with *Bam*HI, and ligated with the 5.9 kb *Bam*HI fragment of pMI569, which contained *C. thermophilum cbh1* CBM, resulting in pMI566. To construct the fusion gene *Tecbh1-HgCBM-C*, the catalytic domain of *Tecbh1* was amplified from the plasmid pMI549 with primers Te-CBH-F and Te-CBH-R, and the *H. grisea cbh1* CBM domain was amplified from the plasmid pRDH103 by primers Hg-CBM-F and Hg-CBM-R. The catalytic domain was digested with *Eco*RI and *Bsm*BI, the CBM domain with *Bsm*BI and *Xho*I, and the plasmid pRDH105 with *Eco*RI and *Xho*I. The three purified fragments were subsequently ligated and transformed into *E. coli*.

For combined expression of the *Tecbh1-TrCBM-C* and *Trcbh2* genes the *PGK1*p*-Trcbh2-PGK1*t unit was excised from pRDH107 with *Bam*HI digestion, the overhangs were filled in with the Klenow enzyme, followed by *Nar*I digestion, and the 3.5 kb fragment was ligated to the 7.7 kb *Pvu*I*-Nar*I fragment of pMI529 generating pMI553 (Table 3). The *ENO1*p*-cbh1-ENO1*t cassette in pMI553 was excised by *Spe*I*-Mlu*I digestion and replaced by the *ENO1*p*-cbh1-ENO1*t cassettes from pRDH103, pRDH105 and pMI569 generating pMI577, pMI578, and pMI579. Subsequently the *PGK1*p*-Trcbh2-PGK1*t unit was replaced by the *PGK1*p*-Clcbh2b-PGK1*t generating pMI580, pMI581, pMI582, and pMI583. For combined expression of *cbh1* and *cbh2* encoding genes on pRDH109, pRDH125, pRDH118, pRDH120, pRDH123, pRDH138, and pRDH140 the entire *ENO1*p*-cbh1-ENO1*t expression cassettes were excised from the relevant plasmid by digestion with *Bam*HI and *Bgl*II and cloned into the unique *Bam*HI restriction site on the relevant *cbh2* containing plasmid (Table 3).

DNA transformation of *S. cerevisiae* Y294 was performed with the lithium acetate dimethylsulfoxide (DMSO) method. [1]. Yeast expression vectors were transformed to *S. cerevisiae* Y294 and transformants were selected on SCD^-URA^ medium. Autoselective strains were constructed by subsequent transformation with pDF1 [6], to ensure maintenance of the *URA3*-bearing expression vectors in complex medium [6; 7]. The presence of the recombinant gene(s) in yeast transformants and the subsequent disruption of the *FUR1* gene to confer autoselectivity were confirmed by PCR or by Southern analysis of *Sca*I-*Xho*I digested DNA using the *FUR1* disruption cassette as a probe (not shown).

**Enzyme assays**

To determine exoglucanase activity on a polymeric substrate, 300 μl of the yeast culture supernatant was added to deep-well microtiter plates with each well containing a 300 μl solution with 2% Avicel PH-105 (FMC Biopolymer, Mechanicsburg, PA, USA), 0.05 M acetate buffer pH 5.0, 0.04% NaN_3_ and 0.3 μl Novozyme-188 (Sigma). The deep-well plate was sealed and incubated on a microtiter plate shaker and shaken at ~1000 rpm at 35°C. 100 μl samples of the enzyme-substrate mixture were taken at the 0 h, 24 h and 48 h time intervals to determine background sugars that were present and the amount of sugars released respectively, using a modified DNS method [8]. The samples were transferred to a 96-well PCR plate using a multi-channel pipette and were centrifuged at 1910 🞨 g for two min. 50 μl of the supernatant were subsequently pipetted along with 100 μl DNS solution into a clean 96-well PCR plate, sealed, heated at 99°C for five min and cooled at 4°C for one min. Absorbance values at 540 nm were read on an X-mark™ (Biorad, Hercules, CA, USA) or on a Varioscan (Thermo Labsystems) microtiter plate reader. Glucose was used to set a standard curve in the range of 0.125 to 4 g/liter from which the amount of glucose released during the assay was determined. The amount of activity was expressed as the percentage of avicel hydrolyzed.

Secreted activities for the strains producing CBH1 (GH7) enzymes were determined using soluble 4-methylumbelliferyl-β-D-lactoside (MULac, Sigma). For the assay 50 μl culture supernatant was added to 50 μL sodium acetate buffer (50 mM, pH 5.0) containing 4.0 mM MULac and incubated at 35°C. Reactions were stopped after 15 minutes of incubation by adding 100 μl 1 M Na_2_CO_3_. Liberation of 4-methylumbelliferone was detected by fluorescence measurement (excitation wavelength = 355 nm, emission wavelength = 460 nm) with a Varian Varioscan spectrofluorometer in a black, flat bottom microtiterplate (Cliniplate, Labsystems). Methylumbelliferone (MU, Sigma) dissolved in 1 M Na_2_CO_3_ was used to set up a standard curve between 0.63 μM and 20 μM. Culture supernatants were diluted as required to keep emission values within this linear range.

**Protein purification**

For larger scale production of the *Te*CBH1-*Tr*CBM-C and *Cl*CBH2b enzymes, 1 or 1.5 liters of YPD medium was inoculated with a 10% volume of an overnight pre-culture of the M0749 strain containing the corresponding expression plasmid. The cultures were grown with shaking (210 rpm) at 35 °C. After 3 days of cultivation the supernatants were harvested by removing the cells by centrifugation. The supernatants were concentrated and changed into 50 mM sodium acetate (pH 5) by ultrafiltration with a 10 kDa cut-off hollow fiber cartridge (GE Lifesciences). The *Te*CBH1-*Tr*CBM-C sample was loaded into DEAE Sepharose FF column equilibrated with 50 mM sodium acetate, pH 5. The bound TeCBH1 was eluted with linear salt gradient of from 0 to 0.5 M NaCl. The elution volumes were 15 and 20 column volumes. The fractions were tested for CBHI activity with MULac by incubating 10µl sample with 90 µl 2 mM MULac in 50 mM NaAc (pH 5), in ambient temperature for 20 minutes and stopping the reaction with 0.5M Na_2_CO_3_. The fluorescence was measured with a Molecular Devices microtiter plate reader (ex. 355 nm and em. 460 nm). The active fractions were pooled then further purified in a second step in which a 5 mL GE phenyl HR column was utilized to further remove media components. In this procedure, the column was equilibrated with 25 mM sodium acetate, 1.2 M ammonium sulfate, pH 5. Ammonium sulfate was added to the sample to bring the concentration in the buffer to 1.2 M and this material was injected onto the column. The protein was eluted with a linear gradient of 25 mM sodium acetate, pH 5 and fractions that were active on MuLac were pooled. The *Te*CBH1-*Tr*CBM-C proteins were visualised on a 4-20% Tris glycine SDS-PAGE and the fractions containing a single band were pooled and buffer exchanged into 50 mM NaAc (pH 5) using 20 ml spin concentrators, cut-off 10 000 Da (Vivaspin, Vivascience GmbH). The protein concentration of the purified preparation was calculated from the absorbance at 280 nm by using the theoretical absorption coefficient calculated from the primary sequence. *Cl*CBH2b was purified using the same chromatography steps as *Te*CBH1-*Tr*CBM-C, DEAE anion exchange followed by phenyl HIC. In this purification, *Cl*CBH2b is found in the flow through of the DEAE step and is eluted from the phenyl HIC column within the decreasing ammonium sulfate gradient. Active fractions were determined using an 1% Avicel hydrolysis assay at pH 5. Active fractions were pooled then purity and concentration were ascertained as described above for *Te*CBH1-*Tr*CBM-C.

**Other protein analysis methods**

Protein concentrations in the cell-free SCD^-URA^ broth were measured with Bio-Rad protein reagent (cat. no. 500-0006) using the Bovine immunoglobulin as the standard (Protein Assay Standard I BioRad 500-0005). Protein concentration in the empty vector control was subtracted from the CBH producing strains to estimate CBH production.

To remove *N*-linked glycans 2.5 μl Endoglycosidase H (Roche, Mannheim, Germany) was added into 20 μl cell-free culture supernatant and incubated overnight according to the manufacturer’s instructions. In control samples, Endoglycosidase H was replaced by water. Protein samples were separated by electrophoresis on precast 12% SDS-PAGE gels (BioRad) and visualized with silver staining.

For determination of the concentration of CBHs produced in bioreactor cultivations by strains expressing *TeCBH1-TrCBM-C* and *ClCBH2b*, a phenyl reverse phase method was developed on an Agilent 2100 HPLC with the MWD detector at 214 and 280 nm. In this method, the purified CBHs described above were used for generating a standard curve from 200 – 10 µg. The sample was injected onto a phenyl RP column (Tosoh phenyl-5PW RP, 4.6 mm x 7.5 cm, 10 µm) that was equilibrated at 55°C in 0.1% trifluoracetic acid (TFA) (w/v), 20% acetonitrile. The protein was eluted from the column at 0.75 ml/min using a linear gradient of acetonitrile with 0.1% TFA (w/v) from 20-60% in 45 minutes. After cleaning the column with 95% acetonitrile/TFA, the column was re-equilibrated. To determine the concentration of *Te*CBH1-*Tr*CBM-C and *Cl*CBH2b produced in media by various strains, the peak area of the sample was compared to the standard curve generated from the peak areas of the purified CBHs (µg/µL injected).

**Determination of plasmid copy number**

Yeast DNA was isolated from cells grown overnight in YPD by phenol extraction from cells broken with glass beads [9] and digested with *Sca*I-*Xho*I. Southern blots were prepared using conventional techniques and hybridized with *URA3* probe labeled with [α-^32^P]dCTP (Perkin Elmer, Boston, MA). Radioactive hybridization signals were detected by scanning exposed storage phosphor screens using the Typhoon 8610 variable mode imager (Molecular Dynamics, Sunnyvale, CA). The signals were quantified using ImageQuant TL 7.0 image analysis software (GE Healthcare). The plasmid copy number expressed in relative units was determined as the ratio between the plasmid-borne and the genomic copy of *URA3.*

**Gene expression studies**

Yeast were grown in 50 ml YPD medium in 250 ml Erlenmeyer flasks at 30°C at 250 rpm and 2 ml samples were removed periodically. Cells were harvested by centrifugation and frozen in dry ice and stored at -70°C. RNA was isolated using the Trizol reagent (Invitrogen cat. no. 15596-018). Northern blots were prepared and hybridized using conventional techniques [1]. Probes for the detection for *T.r.cbh1 CBM, HAC1, ACT1, KAR2* and *PDI1* transcripts were obtained by PCR using sequence specific primers (Additional file 6). The *T.e.cbh1* probe was the 447 bp *Age*I-*Bst*EII fragment from pRDH105, the *T.r.cbh2* probe was the 418 bp *Msc*I-*Xcm*I fragment from pRDH107, and the *C.l.cbh2b* probe was obtained as the 384 bp *Nsi*I-*Stu*I fragment from the corresponding gene synthesized by CodonDevices. Radioactive hybridization signals were detected by scanning exposed storage phosphor screens using the Typhoon 8610 variable mode imager (Molecular Dynamics, Sunnyvale, CA). The signals were quantified using ImageQuant TL 7.0 image analysis software (GE Healthcare).

**Bioreactor propagation of CBH producing yeast strains**

Yeast strains M0759 and M0969 (Tables 1 and 2) were taken from -80°C freezer stocks, grown on YPD media agar plates and inoculated into two 250 ml shake flasks containing 100 ml YPD. The reactor (Biostat A+ with a 2L working volume) was autoclaved for 90 min at 121˚C, and filter sterilized media was added aseptically. Media for the cultivation contained (per liter): 10 g Yeast Extract, 15 g Peptone, 6.7 g Yeast Nitrogen Base without amino acids, 10 ml Vitamin Solution [5 g/liter Nicotinic (folic) acid, 6 g/liter Pyridoxine (vitamin B6), 5 g/liter Thiamine hydrochloride, 0.4 g/liter Biotin], 5 ml Trace Element Solution (10 g/liter ZnSO_4_*H_2_O, 0.2 g/liter CuSO_4_, 0.2 g/liter MnCl_2_*4H_2_O, 0.2 g/liter Na_2_MoO_4_*2H_2_O, 6.2 g/liter Mg_2_SO_4_), 6 drops of antifoam 204 from a 1 ml syringe fitted with a 23 gauge needle, and 50 g glucose. Filter sterilized glucose feed solution contained (per 500 g): 250 g glucose, 3.4 g Yeast Nitrogen Base without amino acids and without ammonium sulphate, 10 ml Vitamin solution (see above), 5 ml Trace Element solution (see above). During fermentation 5 M NH_4_OH was used to control pH and antifoam 204 was used to control foaming. The reactor was controlled at the following set points: pH 5.25, air flow rate: 1.5 liters/min, agitation: 800 rpm, and temperature: 30˚C. The reactor was inoculated with 100 ml of preculture grown as noted above and run in batch mode until the DO spiked twice. Glucose feeding started at 1.4 g/min (0-2 h) and continued at 1.5 g/min (2-6 h), 1.8 g/min (6-10 h), 2.5 g/min (10-24 h). Samples were taken for protein concentration determination by the HPLC method described above, and dry cell weight was determined by LMA analysis following manufacturer instructions (LMA 200PM-000US Microwave Moisture Analyzer, Sartorius).

**Avicel fermentation to ethanol**

The strains *Sc*[*Tecbh1-TrCBM-C*]*,* *Sc*[*C.l.cbh2b*], *Sc*[*Tecbh1-TrCBM-C & Clcbh2b*] and a reference strain expressing no cellulases were grown in 50 ml YPD medium in baffled 250 ml shake flasks incubated at 30°C on a rotary shaker at 200 rpm for 4 days. Subsequently, 25 ml of each culture was added to McCartney bottles containing 0.5 g of Avicel PH-105 to attain a concentration of 20 g/liter (*i.e.* 2 bottles for each culture). In addition, 100 μL the commercially available β-glucosidase preparation Novozyme 188 (Sigma) was added into half of the bottles so that for each strain there was a bottle with and without added enzyme. Subsequently the bottles were sealed with rubber lined caps to maintain the cultures anaerobically and stirred on magnetic stirrers for 7 days. Samples were taken on days 0, 2, 4 and 7 and cellobiose, glucose and ethanol content was determined with high performance liquid chromatography (HPLC). Samples were separated on a Phenomenex Rezex column (RHM-monosacharide H+8% 300x7.8mm) with guard column (security guard KJO-4282) using a Finnigan Surveyor LC pump (Thermo Scientific) and were detected with a Finnigan Surveyor RI detector (Thermo Scientific). The mobile phase used was 5 mM H_2_SO_4_ at a flow rate of 0.7 ml/min at 65°C. For each strain the conversion test was set up in triplicate for both the samples with and without added Novozyme 188.

**References in the Additional material**

1. Sambrook J, Russell DW: *Molecular cloning : a laboratory manual.* Cold Spring Harbor Laboratory Press 2001.
2. Carbone A, Zinovyev A, Kepes F: **Codon adaptation index as a measure of dominating codon bias.** *Bioinformatics* 2003, **19**: 2005-2015.
3. Den Haan RS, Rose H, Lynd LR, Van Zyl WH: **Hydrolysis and fermentation of amorphous cellulose by recombinant *Saccharomyces cerevisiae*.** *Metab Eng* 2007, **9**:87-94.
4. Crous JM, Pretorius IS, Van Zyl WH: **Cloning and expression of an *Aspergillus kawachii* endo-1,4-β-xylanase gene in *Saccharomyces cerevisiae*.** *Curr Genet* 1995, **28**:467-473.
5. Hill J, Ian KA, Donald G, Griffiths DE: **DMSO-enhanced whole cell yeast transformation.** *Nucleic Acids Res* 1991, **19**:5791.
6. La Grange DC, Pretorius IS, Van Zyl WH: **Expression of a *Trichoderma reesei* β-xylanase gene (*XYN2*) in *Saccharomyces cerevisiae*.** *Appl Environ Microbiol* 1996, **62**:1036-1044.
7. Kern L, de Montigny J, Jund R, Lacroute F: **The *FUR1* gene of *Saccharomyces cerevisiae*: cloning, structure and expression of wild-type and mutant alleles.** *Gene* 1990, **88**:149-157.
8. Miller GL, Blum R, Glennon WE, Burton AL: **Measurement of carboxymethyl cellulase activity.** *Anal Biochem* 1960, **2**:127-132.
9. Hoffman CS, Winston F: **A 10-minute DNA preparation from yeast efficiently releases autonomous plasmids for transformation of *Escherichia coli.*** *Gene* 1987, **57**:267-272.
10. Saloheimo M, Lund M, Penttilä ME: **The protein disulphide isomerase gene of the fungus *Trichoderma reesei* is induced by endoplasmic reticulum stress and regulated by the carbon source.** *Mol Gen Genet* 1999, **262**:35-45.
