# Supplementary material for: High level secretion of cellobiohydrolases by Saccharomyces cerevisiae
Source: Biotechnol Biofuels. 2011 Sep 12;4:30. doi: 10.1186/1754-6834-4-30 (PMC3224389; doi:10.1186/1754-6834-4-30)
Supplement: Additional file 6 — Oligonucleotides and restriction fragments used for preparation of probes. This table identifies the nucleotide sequences of the probes used in the Northern hybridizations. [file 1754-6834-4-30-S6.PDF]

Additional file 6. Oligonucleotides and restriction fragments used for preparation of probes.

Probe	PCR primers and restriction fragments	Template
HAC1	627 ScHAC1 fwd-1 CCCACCTACGACAACAACCGCCACT<br>628 ScHAC1 rev+660 CCTATGGATTACGCCAATTGTCAAG	pMS109 [11]
KAR2	629 ScKAR2fwd+212 AGACTGAAATTCTTGCTAATGAGC<br>630 ScKAR2rev+596 GCGTCATTGAAATAAGCAGGAAC	*S. cerevisiae*
PDI1	631 ScPDI1fwd+500 TAAGATTGACGCCGACTTCAAC<br>632 ScPDI1rev+900 TTGAGGCAAACCGTACTTCAAG	*S. cerevisiae*
ACT1	586 ACT1+50F ATGTGTAAAGCCGGTTTTGCC<br>587 ACT1+500R AGCGGTTTGCATTTCTTGTTC	*S. cerevisiae*
CBM+ENO	399Trcbh1 synt CBM5 MlyIHincII<br>GCGACGAGTCAACCCTCCAGGTGGTAACAGAGGTACTACCAC<br>410 ScENO1 term rev GAGCGGGCGGATACACGCGTC	pMI529 (this work)
T.e.cbh1	447 bp *Age*I-*Bst*EII fragment	pRDH105 (this work)
T.r.cbh2	418 bp *Msc*I-*Xcm*I fragment	pRDH107 (this work)
C.l.cbh2b	384 bp *Nsi*I-*Stu*I fragment	pMI574 (this work)
